# Depletion of *M*. *tuberculosis* GlmU from Infected Murine Lungs Effects the Clearance of the Pathogen

**DOI:** 10.1371/journal.ppat.1005235

**Published:** 2015-10-21

**Authors:** Vijay Soni, Sandeep Upadhayay, Priyanka Suryadevara, Ganesh Samla, Archana Singh, Perumal Yogeeswari, Dharmarajan Sriram, Vinay Kumar Nandicoori

**Affiliations:** 1 National Institute of Immunology, New Delhi, India; 2 Department of Pharmacy, Birla Institute of Technology and Science-Pilani, Hyderabad Campus, Hyderabad, India; 3 CSIR-Institute of Genomics and Integrative Biology, New Delhi, India; University of Massachusetts Medical School, UNITED STATES

## Abstract

*M*. *tuberculosis* N-acetyl-glucosamine-1-phosphate uridyltransferase (GlmU_Mtb_) is a bi-functional enzyme engaged in the synthesis of two metabolic intermediates N-acetylglucosamine-1-phosphate (GlcNAc-1-P) and UDP-GlcNAc, catalyzed by the C- and N-terminal domains respectively. UDP-GlcNAc is a key metabolite essential for the synthesis of peptidoglycan, disaccharide linker, arabinogalactan and mycothiols. While *glmU*_*Mtb*_ was predicted to be an essential gene, till date the role of GlmU_Mtb_ in modulating the *in vitro* growth of *Mtb* or its role in survival of pathogen *ex vivo* / *in vivo* have not been deciphered. Here we present the results of a comprehensive study dissecting the role of GlmU_Mtb_ in arbitrating the survival of the pathogen both *in vitro* and *in vivo*. We find that absence of GlmU_Mtb_ leads to extensive perturbation of bacterial morphology and substantial reduction in cell wall thickness under normoxic as well as hypoxic conditions. Complementation studies show that the acetyl- and uridyl- transferase activities of GlmU_Mtb_ are independently essential for bacterial survival *in vitro*, and GlmU_Mtb_ is also found to be essential for mycobacterial survival in THP-1 cells as well as in guinea pigs. Depletion of GlmU_Mtb_ from infected murine lungs, four weeks post infection, led to significant reduction in the bacillary load. The administration of Oxa33, a novel oxazolidine derivative that specifically inhibits GlmU_Mtb_, to infected mice resulted in significant decrease in the bacillary load. Thus our study establishes GlmU_Mtb_ as a strong candidate for intervention measures against established tuberculosis infections.

## Author Summary

The synthesis of the *Mtb* cell wall involves a cascade of reactions catalyzed by cytosolic and cell membrane-bound enzymes. The reaction catalyzed by GlmU_Mtb_ (an enzyme with acetyltransferase and uridyltransferase activities) generates UDP-GlcNAc, a central nucleotide-sugar building block of the cell wall. Apart from cell wall synthesis UDP-GlcNAc is an essential metabolite participating in other cellular processes including disaccharide linker and mycothiol biosynthesis. GlmU_Mtb_ shares very little sequence similarity with eukaryotic acetyltransferase and uridyltransferase enzymes. Many pathogens have alternative pathway(s) for foraging GlcNAc from the host. The present study was undertaken to see the effects of depleting GlmU_Mtb_ on pathogen survival in the host animal. We have generated a conditional gene replacement mutant of *glmU*_*Mtb*_ and find that depletion of GlmU_Mtb_ at any stage of bacterial growth or in mice infected with *Mtb* including a well-established infection, results in irreversible bacterial death due to perturbation of cell wall synthesis. We have developed a novel anti-GlmU_Mtb_ inhibitor (Oxa33), identified its binding site on GlmU_Mtb_, and shown its specificity for GlmU_Mtb_. The study demonstrates that GlmU_Mtb_ is a promising target for therapeutic intervention and Oxa33 can be pursued as a lead molecule.

## Introduction

The cell wall, which contains a number of virulence determinants, is the first line of defence for survival of the pathogen in the hostile host environment [1]. The mycobacterial cell envelope includes three layers of cell membrane and a cell wall made up of peptidoglycan, mycolic acid, arabinogalactan and lipoarabinomannan (LAM) [2–4]. Most existing first line and second line drugs used to treat TB such as isoniazid, ethambutol, ethionamide and cycloserine, act on enzymes engaged in the synthesis of different cell wall components [5]. The current high mortality rates of infected individuals as well as increasing incidence of multidrug-resistant (MDR) and extensively drug-resistant (XDR) tuberculosis (TB) among patients underscore the importance of finding new targets for therapeutic intervention.

**Fig 1:**
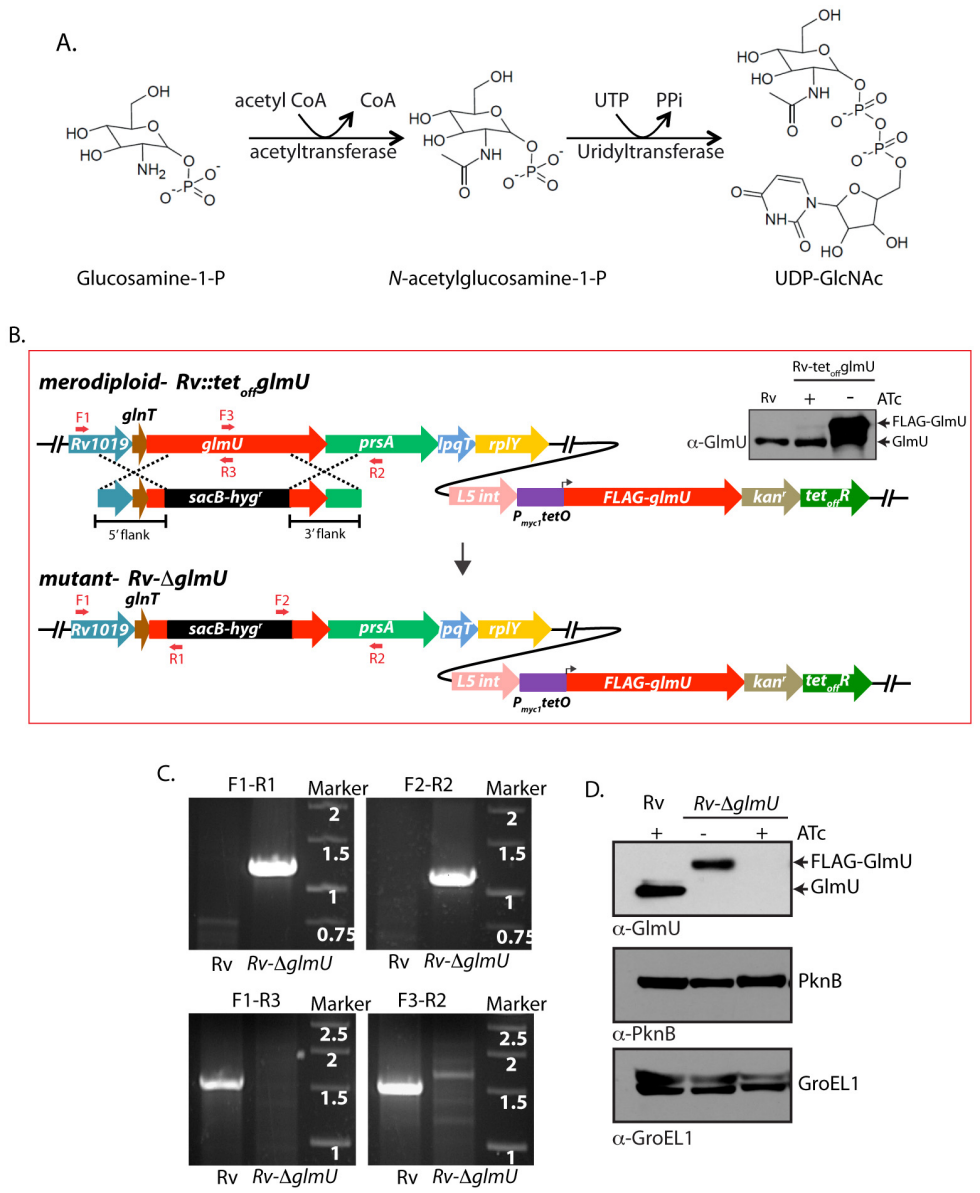
Generation of *glmU*_*Mtb*_ conditional deletion mutant. (**A**) Schematic depicting the biochemical reaction catalyzed by GlmU_Mtb_. (**B)** Schematic diagram representing the genomic location of *glmU*_*Mtb*_ (*Rv1018c*) and homologous recombination between flanking sequence in the phagemid and the genomic locus. Primers used for the PCR amplification are depicted. Upper right panel shows the immunoblot. WCLs from *Rv* or *Rv*::*glmU* grown in the presence or absence of ATc were resolved and probed with anti-GlmU antibodies. Bands corresponding to the endogenous GlmU and ectopic FLAG-GlmU are indicated. (**C)** Agarose gel showing the PCR amplification of the *Rv* & putative *Rv*∆*glmU* mutant using specific primers. Primers F1 and R2 are beyond the flanks, R1 and F2 belong to resolvase sites in *sacB/hyg* ^*R*^ cassette and F3 and R3 binds to the native *glmU*. Amplification with F1-R1 or F2-R2 primers results in 1.23 kb or 1.17 kb size products with *Rv*∆*glmU* strain but none with the *Rv*. Whereas PCRs with F1-R3 or F3-R2 primers amplifies 1.5 kb band with the *Rv* and none with the *Rv*∆*glmU* mutant. **(D)** Whole cell lysates (WCL) were prepared from the large scale cultures of *Rv* and *Rv*∆*glmU* grown in the absence and presence of ATc for five days. 20 μg of WCLs were resolved and probed with anti-GlmU_Mtb_, anti-PknB and anti-GroEL1 antibodies. Band corresponding to endogenous GlmU_Mtb_ and FLAG-GlmU_Mtb_ are indicated.

GlmU_Mtb_ is a bi-functional enzyme, with acetyltransferase and uridyltransferase activities catalyzed by the C- and N- terminal domains respectively (Fig 1A) [6,7]. The carboxy-terminal domain of GlmU_Mtb_ transfers the acetyl moiety from acetyl CoA onto glucosamine-1-phosphate to generate N-acetylglucosamine-1-phosphate (GlcNAc-1-P). The N-terminal uridyltransferase domain of GlmU_Mtb_ then catalyzes the transfer of UMP (from UTP) to GlcNAc-1-P to form UDP-GlcNAc (Fig 1A) [6]. The UDP-GlcNAc thus produced is among the central metabolites that is required for the synthesis of peptidoglycan, lipid A of LAM, arabinogalactan, Rha-GlcNAc linkers, mycothiol (required for maintaining redox homeostasis) [8–14]. The crystal structure of *M*. *tuberculosis* GlmU (GlmU_Mtb_) displays two-domain architecture with an N-terminal α/β- like fold and a C-terminal left-handed parallel-β-helix structure [15,16]. Unlike its orthologs, GlmU_Mtb_ has a long carboxy-terminal tail which displays little secondary structure [17]. Results from transposon mutagenesis experiments have indicated *glmU*_*Mtb*_ to be an essential gene, supported by the fact that *M*. *smegmatis* is unable to grow in the absence of *glmU*_*smeg*_ [18–20]. However, no studies have addressed the question of whether both the activities of GlmU_Mtb_ are independently essential for the growth or survival of the bacterium.

While the enzymes required for the synthesis of UDP-GlcNAc are well conserved among prokaryotes, they are very different from those found in eukaryotes, making GlmU_Mtb_ an attractive putative drug target [21,22]. Researchers have developed compounds that inhibit the activities of the orthologs of GlmU_Mtb_ (GlmU from *T*. *brucei*, *P*. *aeruginosa*, *E*. *coli*, *H*. *influenza* and *X*. *oryzae) in vitro* [23–30]. Bioinformatic analyses and kinetic modelling data advocate GlmU_Mtb_ to be a potential target for the development of suitable inhibitors [31]. In concurrence with these predictions, effective inhibitors have been developed against, the acetyltransferase and uridyltransferase domains of GlmU_Mtb_ [32,33]. However, the precise sites of inhibitor-protein interactions and the efficacy of the inhibitors *ex vivo* or *in vivo* have not been investigated. Subjecting *Mtb* cultures *in vitro* to gradual decrease of oxygen (hypoxic stress) results in reprogramming of metabolic pathways and up-regulation of stress response genes, and is considered to be an *in vitro* model for the dormancy [34,35]. The importance of GlmU_Mtb_ for growth under hypoxic conditions and in an *in vivo* infection model is yet to be investigated. In the present study we have generated a conditional gene replacement mutant of *glmU*_*Mtb*_ and used this mutant to investigate any role GlmU_Mtb_ may play in modulating the growth of the bacterium *in vitro*, *ex vivo* and *in vivo*. The data presented here demonstrate that GlmU_Mtb_ is a viable and promising target for therapeutic intervention against tuberculosis.

## Results

### GlmU_Mtb_ depletion perturbs cell wall structure and affects the bacterial survival in normoxia

**Fig 2:**
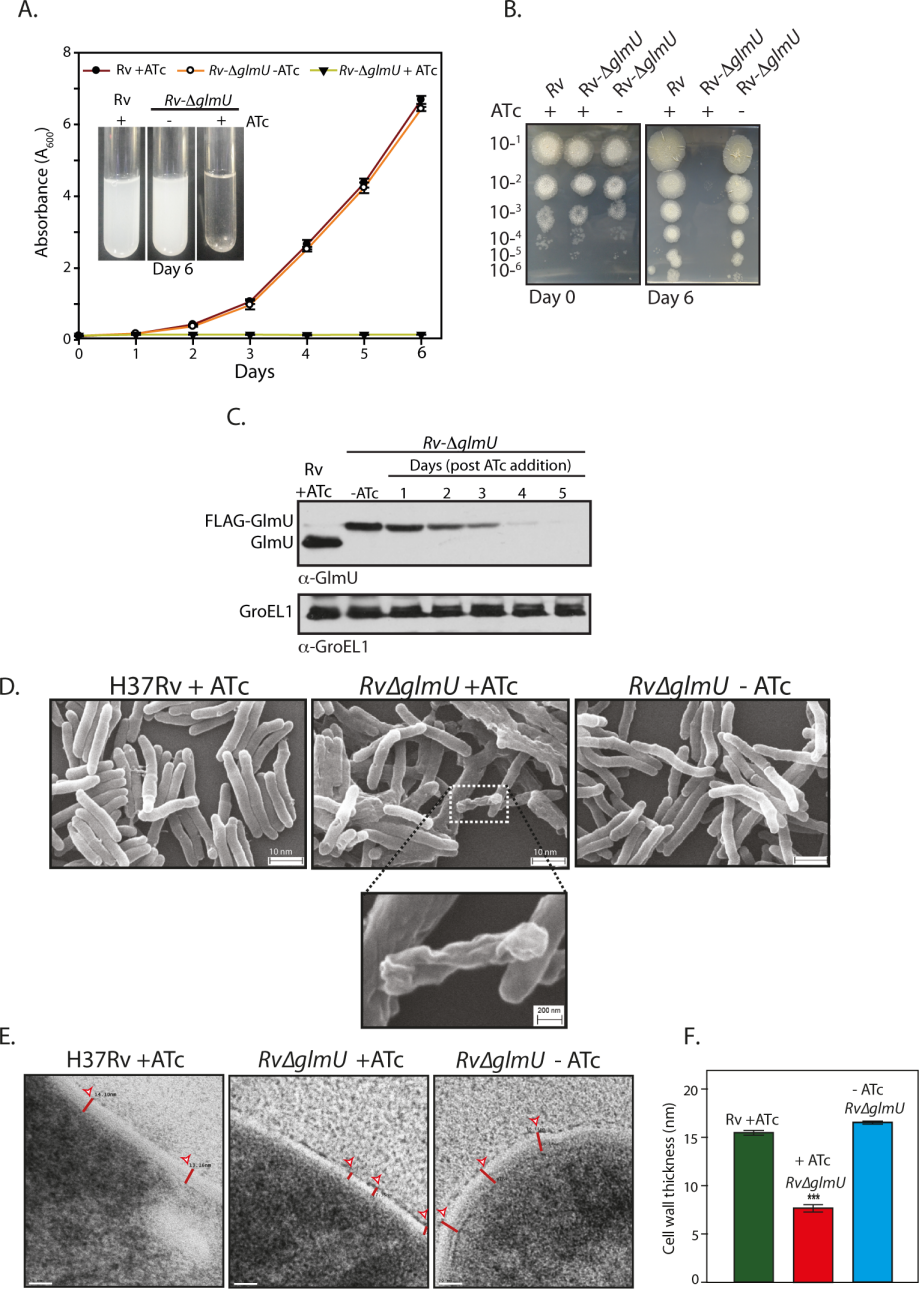
GlmU_Mtb_ depletion affects the bacterial survival by perturbing the cell wall structure. **(A)** *Rv* and *Rv*∆*glmU* cultures grown to an *A*_*600*_ of 0.8 were used to seed fresh cultures at an initial *A*_600_ of 0.1 in the presence or absence of ATc as indicated and the day wise growth was monitored for six days. Inset shows the growth in the culture tubes after six days. The experiment was performed in triplicates (n = 3). Error bars indicate standard error of mean (s.e.m). **(B)** Day 0 and Day 6 cultures grown in the presence or absence of ATc were serially diluted and spotted on 7H11 agar plates. **(C)** Large scale cultures were inoculated at A_600_ of 0.1 in the presence or absence of ATc and the WCLs were prepared on different days post ATc addition (as indicated). WCLs were resolved and probed with anti-GlmU_Mtb_ and anti-GroEL1 antibodies. **(D)** Scanning electron microscopy of *Rv* and *Rv*∆*glmU* grown for 72 h with or without ATc as indicated. Scale bars in upper panel were 10 nm and for the inset was 200 nm. The experiment was repeated thrice. **(E)** Transmission electron micrographs results at 50,000X of *Rv* and *Rv*∆*glmU* cultures grown with or without ATc. Red lines depict the cell wall thickness. Scale bar: 20 nm. **(F)** Cell wall thickness was measured in nm for 15 to 22 cells for each sample. ***p<0.0001, two tailed non parametric *t*-test, mean, s.e.m.

As the tetracycline-inducible system is an effective means to regulate gene expression [36], we introduced the integration-proficient pST-KirT-*glmU* construct (wherein *glmU*_*Mtb*_ gene was cloned under a promoter that shuts down upon ATc addition; S1A Fig) into *Mtb H37Rv* (Fig 1B). Whereas the expression of GlmU_Mtb_ from its native locus remained unaltered, the expression of FLAG-GlmU_Mtb_ in *Rv*::*glmU* strain was drastically compromised in the presence of ATc (Western blot inset, Fig 1B). This merodiploid strain was transduced with temperature sensitive phage, and the fidelity of homologous recombination at the native locus was confirmed by amplification across the replacement junctions using appropriate primers (Fig 1C). A comparison of GlmU_Mtb_ expression in the presence and absence of ATc revealed that the protein was not detectable by western blot analysis after 6 days of growth in the presence of ATc (Fig 1D). While the growth of *Rv*∆*glmU* in the absence of ATc was similar to *Rv*, in the presence of ATc the growth was drastically compromised (Fig 2A). A comparative analysis of growth by spotting of serially diluted cultures of *Rv* and *Rv*∆*glmU* grown in the presence versus absence of ATc showed that GlmU_Mtb_ depletion by addition of ATc led to complete inhibition of growth, with no growth detected after 6 days (Fig 2B). Interestingly, analysis of GlmU_Mtb_ expression every 24 hours post-ATc addition uncovered significant reduction in GlmU_Mtb_ expression by the third day itself (Fig 2C). These results indicate that GlmU_Mtb_ is required for the *Mtb* survival. To determine the impact of GlmU_Mtb_ depletion on cellular morphology we carried out SEM and TEM imaging analysis of *Rv and Rv*∆*glmU* cells grown for three days in the absence or presence of ATc. SEM analysis revealed severe morphological perturbations in the absence of GlmU_Mtb_, with the bacilli showing wrinkled surface and fused cells (Fig 2D). TEM analysis showed that whereas in *Rv* and *Rv*∆*glmU* cell wall structure and thickness are comparable, there was a marked decrease in cell wall thickness in *Rv*∆*glmU* cells where GlmU_Mtb_ was not expressed (cells grown in the presence of ATc; Fig 2E and 2F).

### Impact of GlmU_Mtb_ depletion on dormant bacteria

**Fig 3:**
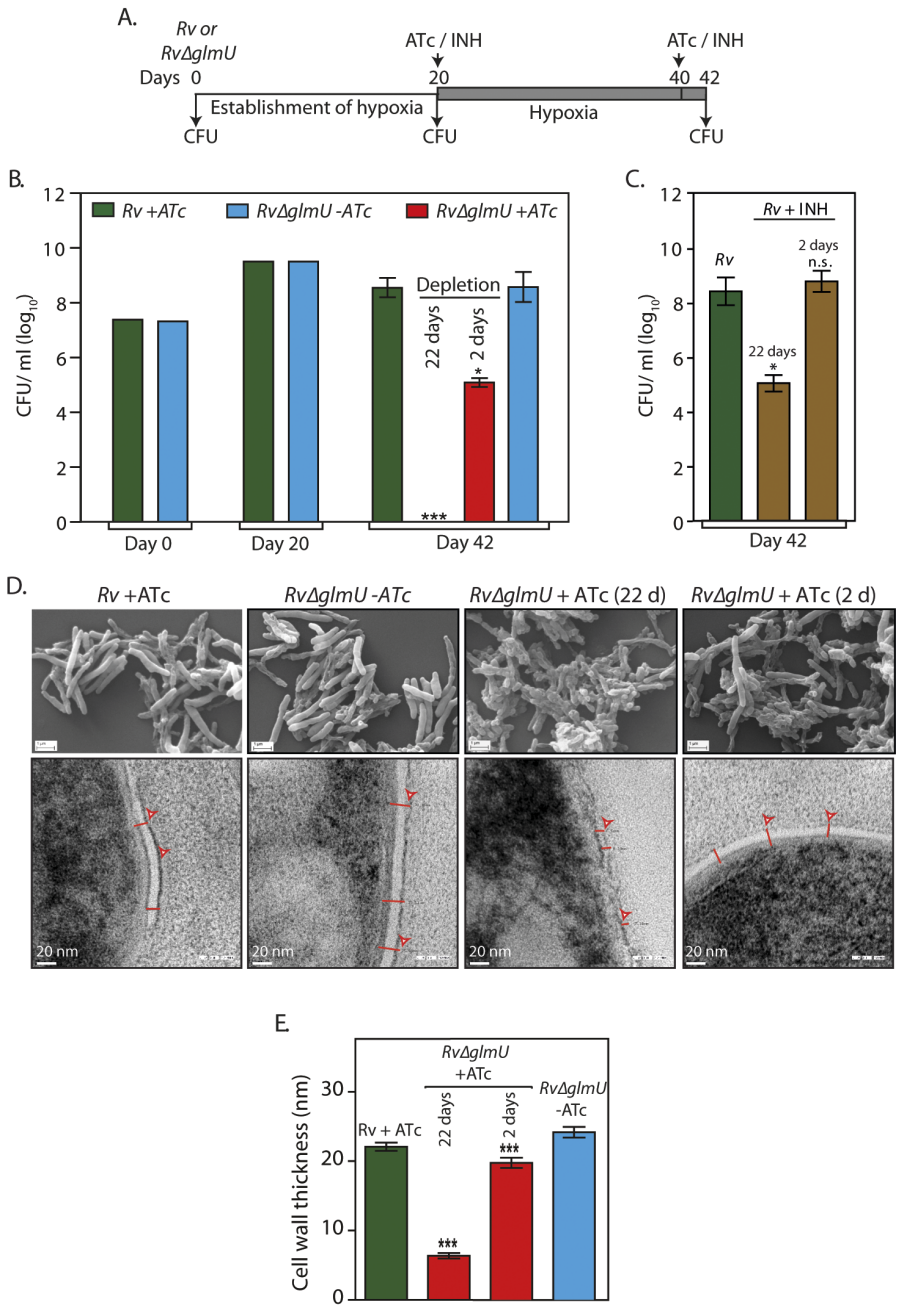
Effect of GlmU_Mtb_ depletion of dormant bacteria. ** (A)** Schematic outline of the experiment. **(B)** *Rv* and *Rv*∆*glmU* cultures were seeded at an initial *A*_600_ of 0.1 in 1.5 HPLC tubes or in 500 ml flasks containing penetrable caps. Establishment of hypoxia was monitored with the help of methylene blue color change (blue to colorless). ATc was added to *Rv* on 20^th^ day and to *Rv*∆*glmU* on 20^th^ and 40^th^ day. CFUs were enumerated on day 0, day 20 and day 42. *p<0.05 or ***p<0.005, two tailed non parametric *t*-test, Error bars indicate s.e.m. and the experiment was performed in triplicates (n = 3). **(C)** INH (50 ng/ml) was added to the hypoxic *Rv* cultures on 20^th^ and 40^th^ day. CFUs were enumerated on day 42. *p<0.05 or NS: non- significant, two tailed non parametric *t*-test, Error bars indicate s.e.m. and n = 3. **(D)** Large scale hypoxic cultures were pelleted down on day 42 and processed for scanning electron microscopy (upper panel) or transmission electron microscopy (lower panel) imaging. (SEM: scale bars: 1 μm; TEM scale bar: 20 nm). Cell wall thickness is indicated by red lines. **(E)** Cell wall thickness was measured in nm for 20 to 54 cells from the TEM images of different samples (representative image shown in Fig 3C). ***p< 0.0001 and 0.0005, two tailed non parametric *t*-test, Error bars indicate s.e.m.

Next we used the Wayne model to investigate the consequence of GlmU_Mtb_ depletion on the dormant bacteria under hypoxic conditions [35]. Accordingly, hypoxia was established and maintained for 42 days with depletion of GlmU_Mtb_ or addition of INH for either 22 days, or for 2 days (Fig 3A, line diagram). In agreement with previous reports, we observed that bacteria were tolerant to INH under hypoxic conditions [37] (Fig 3C), with a thicker cell wall being observed under hypoxic conditions compared with the normoxic cultures (Fig 3D and 3E). Depletion of GlmU_Mtb_ for 22 days resulted in complete clearance of growth (Fig 3B), which was also reflected in severe morphological perturbations and drastic reduction in cell wall thickness (Fig 3D and 3E). Significantly, GlmU_Mtb_ depletion for as less as 2 days decreased cell viability by three orders of magnitude (Fig 3B) and decrease in cell wall thickness (~18%; Fig 3D and 3E). Taken together, the data suggests that the absence of GlmU_Mtb_ in hypoxic condition leads to aberrant cell wall thickness and morphology, eventually leading to the death of the cell.

### Acetyl and uridyltransferase activities of GlmU_Mtb_ are independently essential

**Fig 4:**
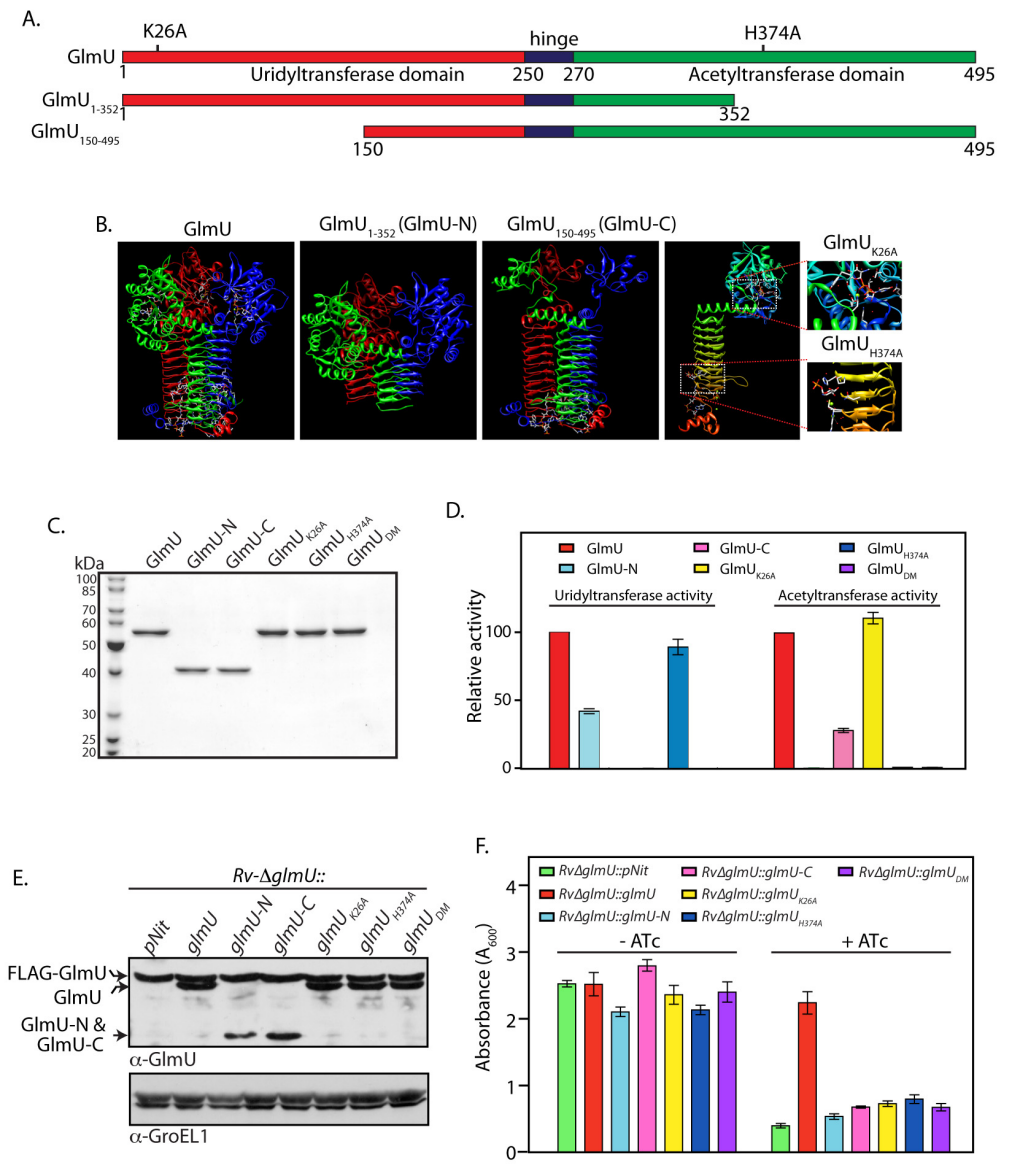
Acetyl and uridyltransferase activities are independently essential. **(A)** and **(B)** Schematic and cartoon representation of GlmU_Mtb_ depicting different domains, active site residues and the deletion mutants. **(C)** GlmU_Mtb_ and GlmU_Mtb_-mutants were purified as described earlier [38] and the 1 μg of the purified proteins were resolved on 10% SDS-PAGE and stained with coomassie. **(D)** Uridyltransferase (left panel) and acetyltransferase (right panel) activities were carried out as describe in Methods using 0.5 to 20 pmoles of wild type or mutant GlmU_Mtb_ proteins. Activity was defined as μM product formed / min / pmole of enzyme. Relative activities of the mutants were calculated with respect to the activity of GlmU_Mtb_, which was normalized to 100%. The experiment was repeated three times and the error bars indicate s.e.m. **(E)** Wild type and mutated GlmU_Mtb_ genes were cloned into pNit vector without any N- or C- terminal tag. pNit-*glmU*_wt_ or pNit-*glmU*_mutant_ constructs were electroporated into *Rv*∆*glmU*, and the WCLs prepared from *Rv*∆*glmU and Rv*∆*glmU*::*glmU*_*mutant*_ cultures were resolved and probed with anti-GlmU and anti-GroEL1 antibodies. Bands corresponding to FLAG-GlmU_Mtb_, complemented GlmU_*wt/mutant*_ and the deletion fragments of GlmU are indicated. **(F)** *Rv*∆*glmU and Rv*∆*glmU*::*glmU*_*mutant*_ cultures were seeded at an initial A_600_ of 0.1 and grown for five days in the absence or presence of ATc. The experiment was performed in triplicates and the error bars represent s.e.m.

Biochemical investigations have shown that the N-terminal fragment (1–352 amino acids) and C-terminal fragment (150–495 amino acids) of GlmU_Mtb_ can independently undertake uridyltransferase and acetyltransferase activities respectively (Fig 4A and 4B) [15,17]. The active site residues that are necessary for these activities have also been identified (Fig 4A and 4B) [17]. To investigate if both activities are essential for cell survival, we have generated previously reported truncation mutants GlmU-N and GlmU-C [38]. We also generated GlmU_K26A_ and GlmU_H374A,_ the uridyltransferase and acetyltransferase active site mutants, and GlmU_DM_ wherein both the active site residues were concomitantly mutated. GlmU_Mtb_ wild type and mutant proteins were purified (Fig 4C) and their uridyltransferase and acetyltransferase activities were assayed. While GlmU-C and GlmU_K26A_ mutants showed acetyltransferase activity, as expected they did not show any uridyltransferase activity (Fig 4D). On the other hand GlmU-N and GlmU_H374A_ had uridyltransferase activity but not the acetyltransferase activity (Fig 4D). As expected the double mutant did not have either uridyl or acetyltransferase activity (Fig 4D). Next complementation assays using one or other truncations / active site mutants were carried out. The FLAG-GlmU_Mtb_ and the complemented untagged wt-GlmU_Mtb_ proteins were found to be expressed at similar levels (Fig 4E). The episomally expressed wt-GlmU_Mtb_ could rescue the *Rv*∆*glmU* phenotype in the presence of ATc (Fig 4F). Contrastingly, while the various GlmU_Mtb_ mutant proteins were expressed at levels comparable to that of FLAG-GlmU_Mtb_ (Fig 4E); none of them rescued the growth defects of the *Rv*∆*glmU* strain in the presence of ATc (Fig 4F). These results indicate that both uridyltransferase and acetyltransferase activities of GlmU_Mtb_ are essential for pathogen survival and imply that the only source of the metabolites GlcNAc-1-P and UDP-GlcNAc is through the GlmU_Mtb_ mediated synthesis pathway.

### Presence of GlmU_Mtb_ is obligatory for the survival of *Mtb* in the host

**Fig 5:**
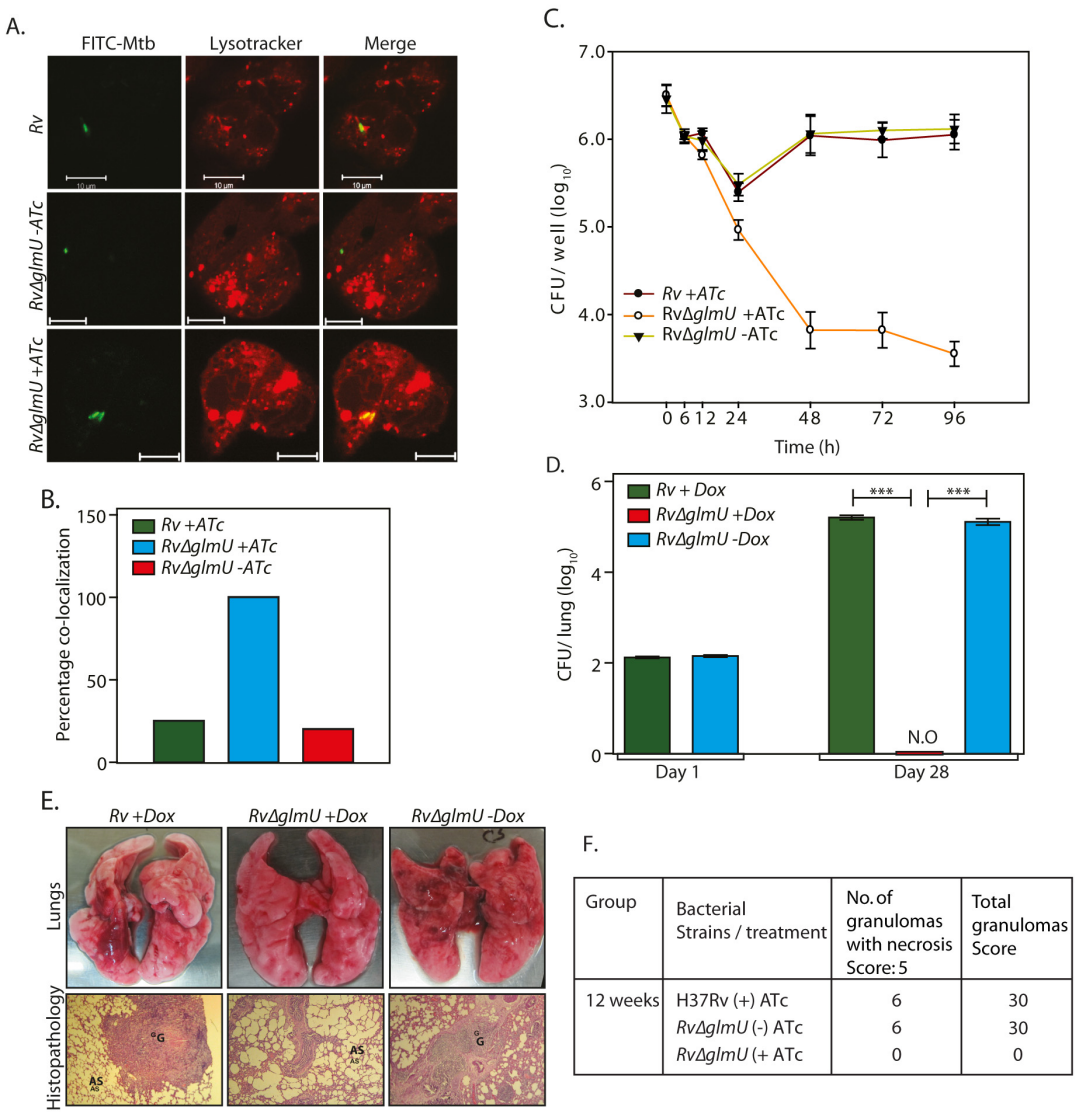
Presence of GlmU_Mtb_ is obligatory for the survival of *Mtb* in the host. **(A)** Confocal microscopy images of THP1 cells infected with *Rv* and *Rv*∆*glmU* in the presence or absence of ATc as indicated were taken 48 h post infection. Bacteria were stained with FITC (green) and lysosomes were stained with Lyso Tracker red DND 99 (red). Scale bars, 10 μm. **(B)** Quantification of percentage co-localization of bacterium and lysosomes in Fig 3A. n = 10 to 15. **(C)** PMA differentiated THP1 cells were infected with *Rv* and *Rv*∆*glmU* and either ATc (400 ng/ml) or the vehicle were added at 0 h. CFUs were enumerated at different time points post infection. The experiment was performed in triplicates and error bars represent s.e.m. **(D)** Guinea pigs were infected with 150–200 bacilli CFUs of *Rv* or *Rv*∆*glmU* and Dox was provided in the water as indicated. Guinea pigs (n = 2) were sacrificed on day 1 and homogenates from the lungs were plated in triplicates to determine the implantation. Guinea pigs (6 guinea pigs /group) were sacrificed four weeks post infection and CFUs were determined in the lung homogenates and results were plotted with log_10_ /lung on the Y-axis and samples on the X-axis. At four weeks post infection mean CFUs for *Rv* +Dox or *Rv*∆*glmU* +Dox and *Rv*∆*glmU*–Dox were 5.2, 0 and 5.11 on log_10_ scale.***p<0.0001, two tailed non parametric *t*-test, error bars represent s.e.m. **(E)** Overall pathology (upper panel) and histopathology (lower panel, HE, 40X) of the infected guinea pig lungs four weeks post infection. Both *Rv* +Dox and *Rv*∆*glmU* -Dox were having prominent granuloma and necrotic lesions in the central and epithelioid cells and lymphocytes around it. Guinea pigs infected with *Rv*∆*glmU* +Dox were showing normal lung parenchyma with clear alveolar spaces. G = Granuloma, AS = Alveolar Space. **(F)** Granuloma scores from histopathology results.

*Mtb* cells devoid of an intact cell wall have been found to be capable of surviving inside the host [39,40]. Some pathogens have been reported to resort to cell wall “recycling” for the synthesis of UDP-GlcNAc, and others have been known to utilize GlcNAc from the host for this purpose [41–44]. However, such mechanisms have not yet been reported in *Mtb*. To investigate these possibilities we examined the impact of GlmU_Mtb_ depletion on survival of the pathogen in the host. Using an *ex vivo* THP-1 infection model we observed ~80% phagolysosome fusion in the absence of GlmU_Mtb_ (Fig 5A and 5B; compare *Rv*∆*glmU* with *Rv*∆*glmU* +ATc). This was also reflected in the survival pattern of the pathogen upon depletion of GlmU_Mtb_ (Fig 5C), with survival being strongly compromised in absence of GlmU_Mtb_. The impact of GlmU_Mtb_ depletion was evident as early as 24 h post-infection, with a dramatic drop in survival by 48 hours post-infection (Fig 5C). The consequences of GlmU_Mtb_ depletion on survival of the pathogen *in vivo* were evaluated using guinea pig infection model. CFUs obtained 24 h after infection suggested efficient and equivalent implantation of both wild type and mutant bacilli in the lungs of guinea pigs (Fig 5D). Discrete bacilli were observed in the lungs of guinea pigs infected with *Rv* and *Rv*∆*glmU* 28 days post-infection (Fig 5E and 5F). In contrast, the lungs of the guinea pigs infected with *Rv*∆*glmU* in the presence of doxycycline were clear (Fig 5E and 5F). In addition splenomegaly was significantly reduced upon depletion of GlmU_Mtb_ (*Rv*∆*glmU* + Dox; S2A and S2B Fig). Whereas the bacillary load in the lungs and spleen of guinea pigs infected with *Rv* and *Rv*∆*glmU* were comparable, we did not detect any bacilli when the *Rv*∆*glmU* infected guinea pigs were administered Dox (Fig 5D). In accordance with these observations, while the gross pathology of lungs infected with *Rv* and *Rv*∆*glmU* displayed considerable granulomatous architecture, normal lung parenchyma was observed upon GlmU_Mtb_ depletion (Fig 5E). These results suggest that the presence of GlmU_Mtb_ is obligatory for mycobacteria to survive in the host.

### Depletion of GlmU_Mtb_ from infected lungs leads to clearance of pathogen

**Fig 6:**
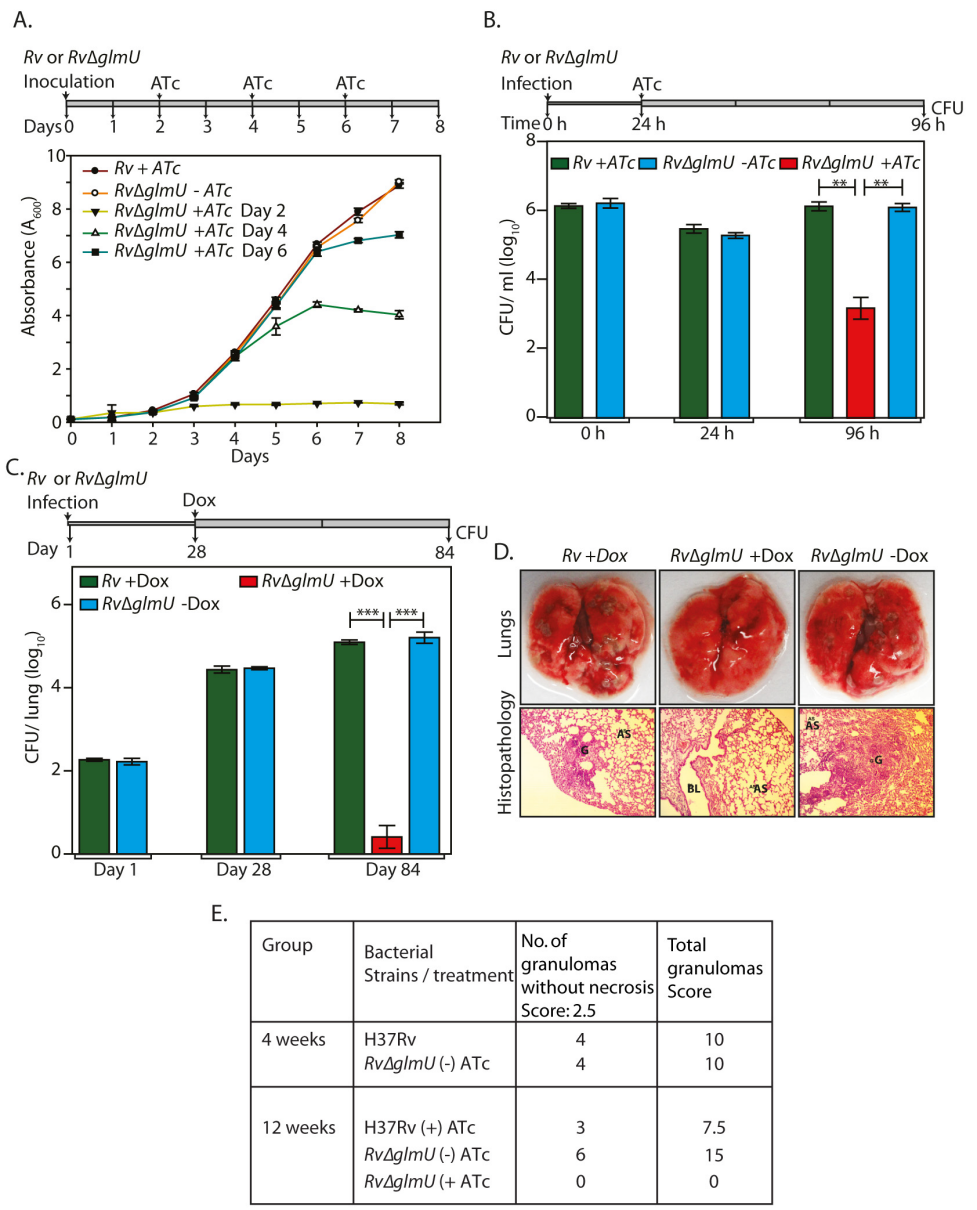
Depletion of GlmU_Mtb_ from infected lungs leads to clearance of pathogen. **(A)** *Rv* and *Rv*∆*glmU* cultures were inoculated at an initial *A*_600_ of 0.1 and the growth was monitored every day for eight days. ATc was added to the *Rv* culture on day 2 and *Rv*∆*glmU* cultures were either grown in the absence of ATc or was supplemented with ATc in the growth media on 2^nd^, 4^th^ or 6^th^ day. Experiment was performed in triplicates and the error bars represent s.e.m. **(B)** Differentiated THP-1 cells were infected *Rv* or *Rv*∆*glmU* cultures and the infection was allowed to be established for 24 h. 24 h post infection ATc was supplemented in the media for *Rv* and *Rv*∆*glmU* infected cultures. As a control THP1 cells infected with *Rv*∆*glmU* were grown without ATc. CFUs were enumerated at 0, 24 and 96 h post infection. **p<0.005, two tailed non parametric *t*-test, Experiment was performed in triplicates and the error bars represent s.e.m. **(C)** BALB/c mice (6 to 9 mice / group) were infected with *Rv* and *Rv*∆*glmU* strains and the infection was established for next 28 days. Subsequently, Dox was provided (with 5% dextrose on every alternate day) in the drinking water for *Rv* infected mice. One group of *Rv*∆*glmU* infected mice were administered with Dox and other with vehicle control for the next 56 days. CFUs were enumerated on day 1, day 28 and day 84 post infection. At 28^th^ days post infection mean CFUs for *Rv* and *Rv*∆*glmU* infected mice were 4.43 and 4.47 on log_10_ scale and 84^th^ days post infection mean CFUs for *Rv* +Dox or *Rv*∆*glmU* +Dox and *Rv*∆*glmU*–Dox were 5.1, 0.42 and 5.2 on log_10_ scale. ***p<0.0005, two tailed non parametric *t*-test, mean, error bars represent s.e.m.. **(D)** Overall pathology and histopathology of infected BALB/c mice lungs. Infected lungs dissected on 84^th^ day after infection from *Rv* (+Dox) and *Rv*∆*glmU* (-Dox) infected mice shows clear lesions (upper panel) and granuloma with lymphocytes and foamy histiocytes (lower panel, HE stain, 100x) while *Rv*∆*glmU* (+Dox) was showing normal lung parenchyma and no granuloma. G = Granuloma, BL = Bronchial Lumen, AS = Alveolar Space. **(E)** Granuloma scores from 4 and 12 weeks of histopathology results.

It was apparent from the data presented above that the addition of ATc or Dox at the time of inoculation or at the time of infection does not allow mycobacterial cell growth or survival in the host. In the ideal candidate for therapeutic intervention, inhibiting the activity of/ depleting the enzyme at any stage of the infection should result in pathogen clearance. We assessed this parameter of GlmU_Mtb_ by providing ATc at different stages of bacterial growth (early, log and stationary phases) and investigating its influence on cell survival in liquid cultures. Addition of ATc to *Rv*∆*glmU* cultures on the 2^nd^, 4^th^ or 6^th^ day after inoculation significantly thwarted growth (Fig 6A). A similar analysis of bacterial growth by serial dilution of cultures followed by spotting on solid medium also revealed that viability was compromised by ~2 log fold 48 h after the addition of ATc, indicating that GlmU_Mtb_ depletion negatively impacted cell survival regardless of which stage of cell growth it was depleted at (S3A Fig).

The influence of GlmU_Mtb_ depletion on an established *ex vivo* infection was estimated by providing ATc 24 h post-infection in a THP-1 infection model. As expected the bacillary load in THP-1 cells infected with *Rv* and *Rv*∆*glmU* were similar at 0 and 24 h after infection (Fig 6B). In contrast, while at 96 h post-infection the bacillary load for *Rv* and *Rv*∆*glmU-* infected THP-1 cells remained the same, the addition of ATc to *Rv*∆*glmU-* infected THP-1 cells 24 h after infection decreased the pathogen load by ~2.5 log fold, indicating that the reduction of GlmU_Mtb_ levels impacts pathogen survival even in an established *ex vivo* infection (Fig 6B). We extended this investigation to analyze the effect of GlmU_Mtb_ depletion from a fully-infected lung using murine infection model. As anticipated, the bacillary load in the lungs of mice infected with *Rv* and *Rv*∆*glmU* were comparable both on Day 1 and on Day 28. Administration of Dox to *Rv*∆*glmU* infected mice for the next 56 days (Day 28 to Day 84) drastically decreased the CFUs in the lungs (Fig 6C) and the pathogen was completely cleared from the spleen (S3B Fig). Unlike the lungs of mice infected with *Rv* and *Rv*∆*glmU*, mice infected with *Rv*∆*glmU* to whom Dox was administered displayed a total absence of lesions and granulomas in the lungs (Fig 6D and 6E). Collectively, these data suggest a fundamental role for UDP-GlcNAc, the end product of the GlmU_Mtb_ -mediated enzymatic reaction, in modulating the persistence of *Mtb* infection.

### Oxa33: A novel allosteric GlmU_Mtb_ inhibitor

**Fig 7:**
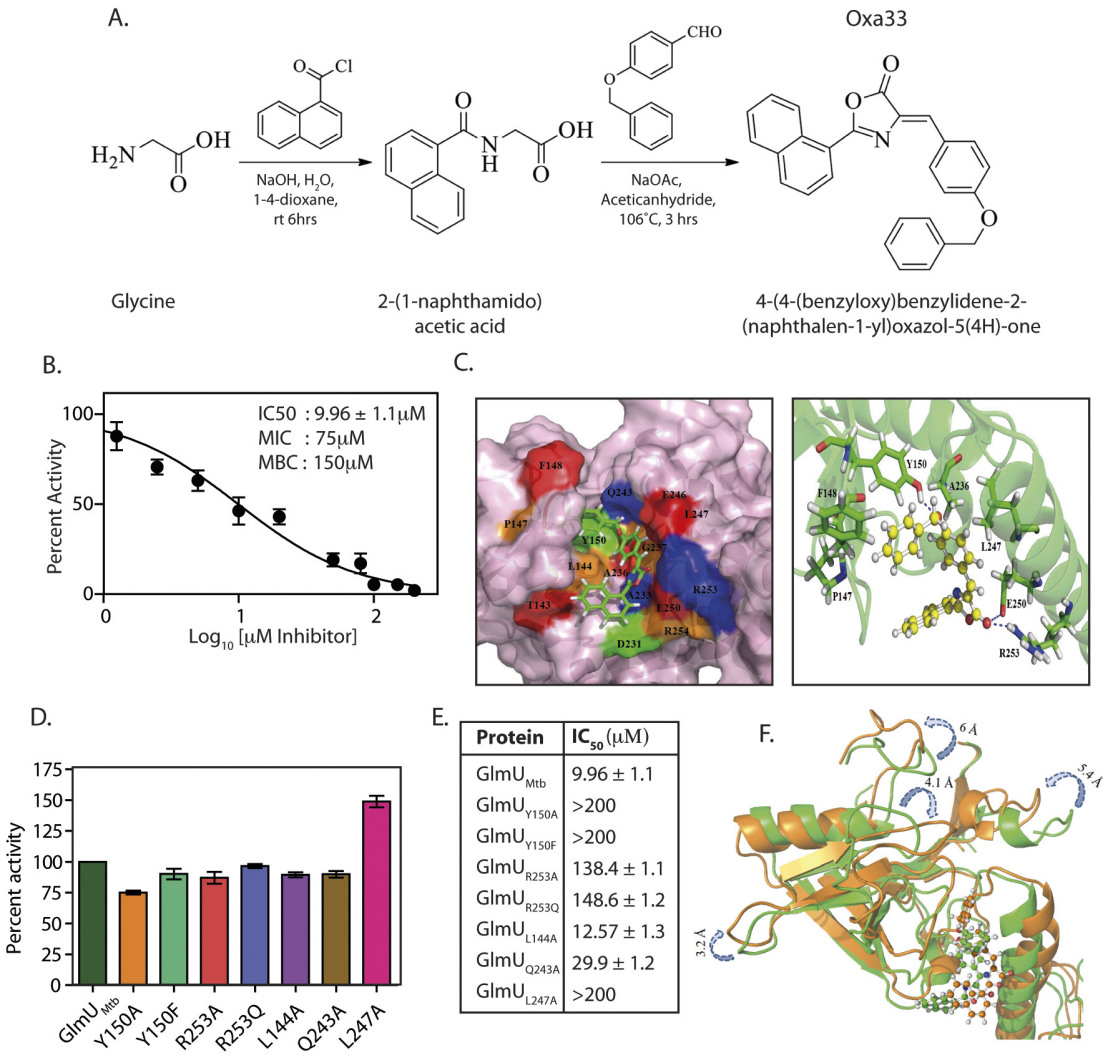
Oxa33, a novel inhibitor against GlmU_Mtb_ binds at allosteric site of uridyltransferase domain. **(A)** Synthetic scheme for the preparation of *4-(4-(benzyloxy)benzylidene)-2-(naphthalen-1-yl)oxazol-5(4H)-one* (Oxa33). **(B)** IC_50_ values were determined by varying the concentration of Oxa33 and calculating the percent activity with respect to the uridyltransferase activity of 0.75 pM conc GlmU_Mtb_ in the presence of 5% DMSO (vehicle). IC_50_ value was calculated by plotting percentage activities against different log_10_ values of inhibitor concentrations (μM). Experiment was performed in triplicates and the error bars represent s.e.m. **(C)** Left panel depicts docking surface representation of the allosteric site on the of the GlmU_Mtb_ allosteric site in complex with Oxa33. Right panel represents ribbon cartoon showing the amino acids residues of allosteric site and hinge region interacting with Oxa33 (in ball and stick model). Tyr150, Glu250 and Arg 253 were found to be in hydrogen bonding with carbonyl oxygen over the oxazole ring. While Leu144, Pro147, Phe148, Tyr150, Ala233, Ala236 and Leu247 participate in strong hydrophobic interactions with Oxa33. **(D)** Based on the data above GlmU_Mtb_ allosteric site mutants were created, proteins were purified and the uridyltransferase activity of GlmU_Mtb_ mutants was determined. Wild type and mutant GlmU_Mtb_ proteins show almost comparable activity. **(E)** IC_50_ values in μM were determined as described above for the wild type and the mutant GlmU_Mtb_ proteins. Tables shows the values obtained for wild type and mutant enzymes. Experiments were performed in triplicates and the error bars represent s.e.m. **(F)** Superimposed view of GlmU_Mtb_-Oxa33 complex before (green) and after (orange) the simulation time period. Showing a partial closure of uridyltransferase site (decreased volume) thereby making it unavailable for its substrates for catalysis.

In addition to the acetyltransferase and uridyltransferase active site pockets, GlmU_Mtb_ also contains an allosteric site. Binding of any suitable molecule/inhibitor to the allosteric site would prevent the conformational change essential for GlmU_Mtb_ uridyltransferase catalytic activity. To target the allosteric site on GlmU_Mtb_ we drew on crystal structure data of *H*. *influenza* GlmU (GlmU_HI_) bound to its allosteric small molecule inhibitor (S4A and S4B Fig) [27]. Alignment of the GlmU_Mtb_ and GlmU_HI_ allosteric pocket residues suggested that the interacting residues were conserved between the two proteins (S4C and S4D Fig). The Asinex database was screened against shape as described (S5A Fig) and the resulting 43 hits were biochemically characterized for their ability to inhibit GlmU_Mtb_ uridyltransferase activity. One of the promising molecules was used for further structural optimization (S5A Fig). Of the 53 structurally optimized compounds one molecule, namely (4*Z*)-4-(4-benzyloxybenzylidene)-2-(naphthalen-2-yl)-1,3-oxazol-5(4*H*)-one (Oxa33; Synthesis scheme provided in Figs 7A and S5B), was found to be an efficient inhibitor of GlmU_Mtb_ activity with an IC_50_ of 9.96±1.1 μM (Fig 7B). Isothermal titration analysis suggested an adequately high affinity binding for the compound (K_a_ = 2.35×10^6^ M^-1^), with a binding stoichiometry of 0.7 (S6A Fig). We sought to identify the residues in GlmU_Mtb_ that are critical for interacting with Oxa33. Docking and MD simulation studies revealed polar, non-polar and hydrophobic interactions between Oxa33 and the allosteric site residues (Figs 7C, S6B and S6C). Based on the obtained data a panel of GlmU_Mtb_ proteins each carrying a single mutation was created, the mutant proteins were purified (S6D Fig), and their uridyltransferase activity assayed. While all the mutants had similar levels of uridyltransferase activity there was a substantial increase in their IC_50_ values, suggesting a loss of interaction with Oxa33 (Fig 7D and 7E).

To decipher the mechanism of Oxa33 mediated inhibition of uridyltransferase activity, we superimposed the GlmU_Mtb_-Oxa33 complex with the unbound GlmU_Mtb_ structure. Upon Oxa33 binding, the loop regions (in the range of 3–6 Å) at the uridyltransferase active site undergo significant conformational changes, decreasing the active site volume, which results in occlusion of the substrates (Fig 7F). Differential scanning fluorimetry (DSF) analysis of GlmU_Mtb_ in the presence of Oxa33 showed a 3°C shift in protein melting temperature (T_m_) validating the conformational changes (S5E Fig). Interestingly we also observed much higher relative fluorescence units (~10000 vs 2500) in the presence of Oxa33, which is likely due to the compound induced structural changes facilitating increased binding of the dye (S6E Fig). Together, these data demonstrate that Oxa33 binds to the allosteric site at N-terminal domain of GlmU_Mtb_ and inhibits its uridyltransferase activity by causing structural changes.

### Specificity and efficacy of Oxa33

**Fig 8:**
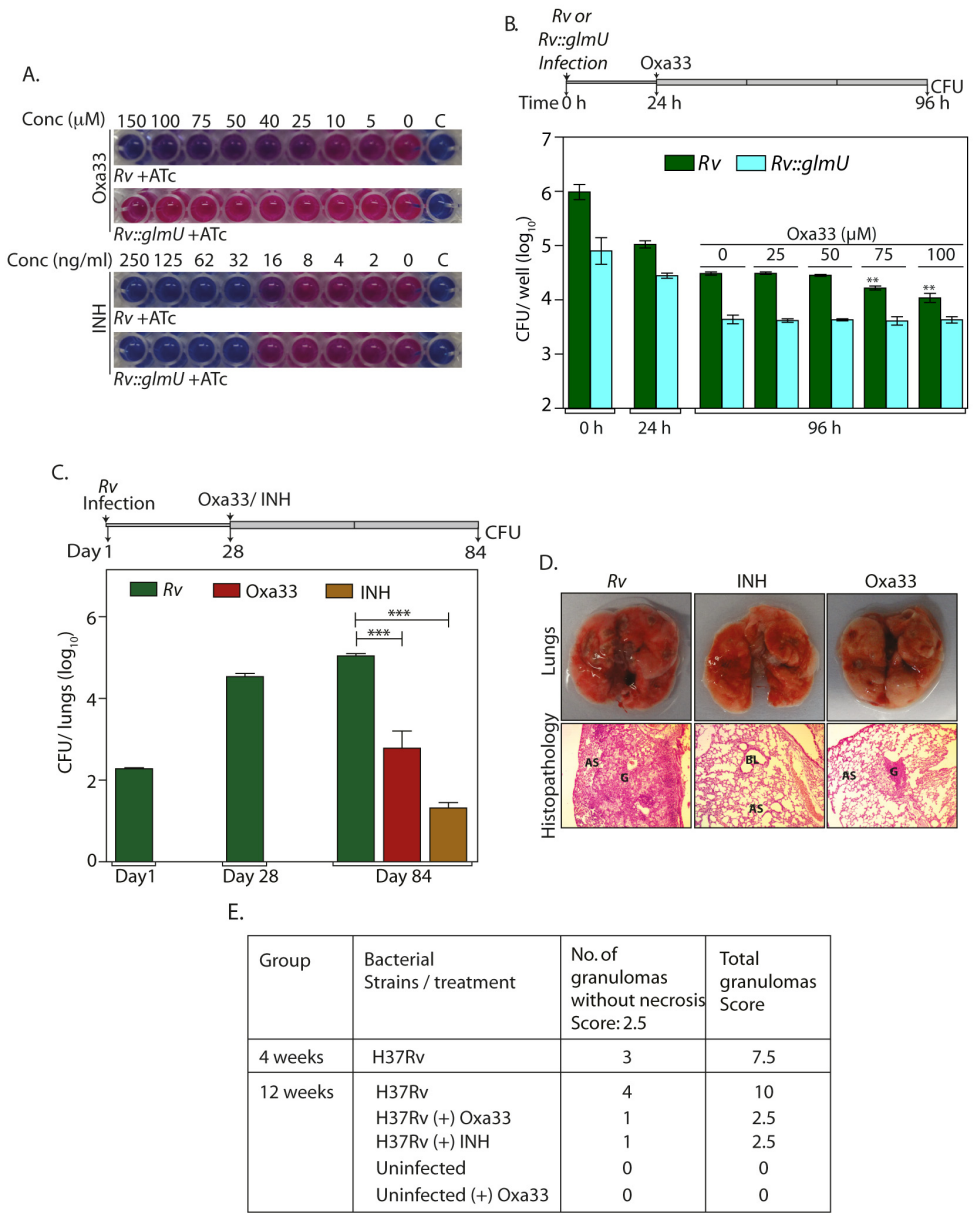
Specificity and efficacy of Oxa33. **(A)** *Rv* and *Rv*::*glmU*_*tet-on*_ cultures were grown in presence of 1 μg/ml ATc and different concentrations of Oxa33 or INH (as indicated). 0.02% resazurin dye was added on day 7. Pink colour indicates viable bacteria while blue colour indicates dead bacteria. ‘C’ denotes culture media control. The experiment was performed thrice. **(B)** Differentiated THP1 were infected either with *Rv* or *Rv*::*glmU*_*tet-on*_ and grown in RPMI media containing 400 ng/ml ATc. 24 h post infection different concentrations (as indicated) of Oxa33 or vehicle control was supplemented to the media for the next 72 h followed by CFU enumeration. Experiment was performed in triplicates and the error bars represent s.e.m. **(C)** BALB/c mice (7 to 11 mice / group) were infected with *Rv* and the infection was allowed to be established for 28 day. Oxa33 (50 mg/kg, intraperitoneal, in 2.5% Tween 80) or INH (25 mg/kg, in drinking water with 5% sucrose) were administered to the mice for a subsequent period of 56 days. CFU in the lungs of mice were determined on 1, 28 and 84 days post infection. Day 1^st^ and day 28^th^ CFUs show successful implantation and establishment of infection, respectively. 56 days of Oxa33 or INH treatment partially reduces the mean bacillary load to 2.78 and 1.3 on log_10_ scale. ***p<0.0005 or 0.0006, two tailed non parametric *t*-test, error bars represent s.e.m. **(D)** While the overall pathology of lungs (upper panel) illustrates robust Mtb infection in untreated mice, relatively smaller and fewer lesions and granulomas were observed in Oxa33 or INH treated mice. Lower panel exhibit hematoxylin and eosin stained photomicrograph of lungs from *Rv*, INH treated and Oxa33 treated mice at 100x magnification. This histopathological analysis shows large or small granuloma with lymphocytes and foamy histiocytes in *Rv* infected mice or Oxa treated mice respectively G = Granuloma, BL = Bronchial Lumen, AS = Alveolar space. **(E)** Granuloma scores from lungs of infected and treated mice for 4 weeks and 12 weeks.

Subsequently we investigated the ability of Oxa33 to inhibit the *in vitro* growth of *Mtb H37Rv*. Oxa33 inhibited the *in vitro* growth of *Mtb H37Rv* with a minimum inhibitory concentration (MIC) of ~75 μM (~30 μg / ml) and a maxium bacteriocidal concentration (MBC) of ~150 μM (~60 μg / ml). To ascertain if this inhibitory effect was due to inhibition of GlmU_Mtb_ activity we overexpressed GlmU_Mtb_ in the cells prior to drug treatment and determined the effect of this on the MIC value (Figs 8A and S6A). Whereas the inhibition of growth in the presence of INH was similar with or without GlmU_Mtb_ overexpression in the cells (Fig 8A, lower panel), Oxa33 failed to inhibit cell growth even at concentrations as high as 150 μM (60 μg/ ml) (Fig 8A, upper panel). Interestingly when sub lethal concentration of Oxa33 was provided, the MIC of INH decreased from 32 to 16 ng/ml (S7B Fig). The impact of Oxa33 on THP1 cells 24 h after infection with either *Rv* or *Rv*::*glmU*_*tet-on*_ was also investigated. In concurrence with the *in vitro* growth data, overexpression of GlmU_Mtb_ alleviated Oxa33-mediated clearance of *Mtb* from THP-1 cells (Figs 8B, S6D and S6E). These results suggest that the inhibition of mycobacterial growth by Oxa33 is specifically due to inhibition of endogenous GlmU_Mtb_.

Finally, we analysed the efficacy of Oxa33 in clearing bacilli from infected lungs using a murine infection model. Oxa33 compound is highly hydrophobic in nature. After trying many solvents, we could successfully resuspend it in 2.5% Tween-80. Prior to performing the experiments we examined the maximum dose tolerance and survival analysis to determine the toxicity (S8A and S8B Fig). Based on the data obtained we chose 50 mg / kg as the appropriate dose. Since it was difficult to predict the fate of Oxa33 during the process of digestion, we avoided using the oral administration route. We chose intra peritoneal route for administering the compound as the intravenous (I/V) injection of Tween 80 (solvent) in the animals was known to cause hypersensitivity and anaphylactic shock [45,46].

Groups of mice were infected with *Rv* and were treated with vehicle, INH, or Oxa33 at 28 days post-infection, for a duration of 56 days (Fig 8C, line diagram). Compared with the vehicle-treated group where we observed a marginal increase in bacillary load, a significant reduction in the bacillary load was observed in the lungs and spleen of both, INH- and Oxa33-treated groups (~4 and 2.5 log fold, respectively for lungs) (Figs 8C and S9A). This was also reflected in the gross pathology and histopathology of lungs (Fig 8D and 8E). Although *in vitro* MBCvalues of Oxa33 was ~150 μM (60 μg/ml), it seems to be a relatively more efficacious *in vivo*, which could be due to its accumulation in the lungs of the infected mice. To investigate this possibility uninfected mice were treated with 50 mg/kg Oxa33 for a period of 3 weeks or 8 weeks. In order to estimate the concentration of Oxa33 in the lung, we first determined the absorbance spectra for Oxa33, which gave a clear peak at 401 nm (S10A Fig). We determined the A_401_ at different concentrations of Oxa33 and the standard curve was plotted (S10B and S10C Fig). Oxa33 was extracted from the lungs and its concentration was determined. The concentrations of Oxa33 in the lungs were in the range of ~200–300 μg /lung at 3 weeks and ~800–1300 μg/lung at 8 weeks (S10D Fig). The accumulation of Oxa33 in the lungs is ~13 to 18 fold higher than the MBC values, which may be the reason for higher potency of Oxa33 *in vivo* compared with the *in vitro* experiments. Taken together, the results presented in this study establish GlmU_Mtb_ to be an effective target against which new sets of inhibitors may be developed.

## Discussion

Cell wall provides the structural rigidity and protects bacteria from various environmental and physiological insults. Biosynthesis of the cell wall of bacteria is a complex process requiring enzymes localized to different cellular compartments [47]. Due to the essentiality of the enzymes involved they are considered attractive targets for anti-microbial therapies. The majority of the first line and second line anti-tuberculosis drugs from the existing regimen target enzymes involved in cell wall synthesis [5]. These include Isoniazid and Ethionamide targeting enoyl-[acyl-carrier-protein] reductase and inhibiting mycolic acid synthesis, Ethambutol targeting arabinosyl transferase and inhibiting arabinogalactan biosynthesis, and Cycloserine targeting D-alanine racemase and ligase, which inhibits peptidoglycan synthesis [5]. However most of these drugs are not very effective against dormant/ latent *Mtb* [48].

UDP-GlcNAc is a critical metabolite both in prokaryotes and eukaryotes. In eukaryotes it is mainly utilized for *O-* or *N-* GlcNAcylation, sialic acid biosynthesis and hylauronic acid biosynthesis [49–51]. In addition to the peptidoglycan synthesis [10], in gram negative bacteria UDP-GlcNAc is required for the synthesis Lipid A subunit of lipopolysaccharide [52] and in gram positive bacteria it is required for Rha-GlcNAc linker [53], arabinogalactan [54], teichioc acid synthesis [55]. In few prokaryotes, UDP-GlcNAc has also been shown to be required for sialic acid [56], *N-*GlcNAcylation [57] and poly (-GlcNAc-)_n_ [58]. GlmU_Mtb,_ an enzyme with dual activity, synthesizes a core metabolite necessary for the synthesis of cell wall peptidoglycan, UDP-GlcNAc [6]. Interestingly, we found that depleting GlmU_Mtb_ during both normoxic and hypoxic growth resulted in substantial decrease in cell viability (Fig 3). This may be due to the requirement of UDP-GlcNAc, which in addition to participating in cell wall synthesis is also required for other cellular processes such as mycothiol biosynthesis (to maintain redox homeostasis) [14,59]. However, the TEM data clearly shows decreased cell envelop thickness even in hypoxic conditions (Fig 3). Although the CFUs do not change significantly the cells may be undergoing significant replication, which might be balanced by death [60]. Alternatively, new cell envelop may be required even if the bacteria are not replicating. Thus one can rule out the possibility that decreased viability may well be due to requirement of UDP-GlcNAc for the cell envelop synthesis.

While UDP-GlcNAc is a critical metabolite for both prokaryotes and eukaryotes, the enzymes involved in its *de novo* synthesis are significantly different [10]. In addition, both prokaryotes and eukaryotes can utilize GlcNAc from different sources to synthesize UDP-GlcNAc through salvage pathways [61–63] (S11 Fig). *Capnocytophaga canimorsus*, a member bacteria from Bacteroidetes phylum lacks endogenous GlmM and GlmU required for the synthesis of GlcNAc and it instead relies on GlcNAc obtained from forages glycans from the host mucin and N-linked glycoproteins [42]. Depending on the enzymes of the salvage pathway present in the bacterial system, it would require either both the activities or only the uridyltransferase activity of GlmU_Mtb_ for UDP-GlcNAc synthesis. Till date the presence of alternate salvage pathway in *Mtb* has not been demonstrated. However, even with an operating salvage pathway GlmU_Mtb_ is essential for the utilization of host GlcNAc to form UDP-GlcNAc (S11 Fig). In line with this, we find that depletion of GlmU_Mtb_ during *ex vivo* or *in vivo* infection either at the start or after infection has been definitively established leads to clearance of pathogen.

GlmU_Mtb_ and the acetyltransferase and uridyltransferase enzymes found in eukaryotes share very little sequence similarity. Although efforts have been made by different groups to target bacterial GlmU proteins, the specificity of these inhibitors for GlmU *in vivo* have not been established [23–30]. Most GlmU inhibitors characterized till date target either the acetyl- or uridyltransferase active sites. In contrast, inhibitors of GlmU_HI_ target the allosteric site near the uridyltransferase active site [27]. The interaction of the inhibitor with the enzyme via this allosteric site perturbs the active site conformation of the protein, thus inhibiting uridyltransferase activity [27]. In the present study we have used shape based designing and developed a novel oxazolidine molecule, Oxa33, and characterized its ability to bind to the GlmU_Mtb_ allosteric site. MD simulation and mutation of critical interacting residues to defined the possible allosteric site residues required for Oxa33 binding (Fig 7). DSF (S6E Fig) and structural superimposition (Fig 7) supports that inhibition of uridyltransferase activity is due to structural changes in the N-terminal domain of GlmU_Mtb_. Further in order to determine the specificity of Oxa33, GlmU_Mtb_ over expressing strains of *Rv* was used to determine the MIC. Both *in vitro* and *ex vivo* results (increased MIC or MBC) validate that Oxa33 specifically binds to GlmU_Mtb_ inside the bacteria. Administrating the Oxa33 to fully infected (28 days) mice resulted in partial ablation of pathogen load in the lungs. Taken together results presented here demonstrates that GlmU_Mtb_ is a viable and promising target for therapeutic intervention and Oxa33 can be pursued as a lead molecule, which needs to be developed further to improve its efficacy.

## Materials and Methods

### Chemicals and reagents

Restriction enzymes and Phu DNA polymerase were purchased from New England Biolabs. pENTR/directional TOPO cloning kit (Invitrogen), pQE2 (Qiagen), were procured from the respective sources. Analytical grade chemicals and oligonucleotide primers were procured from Sigma. Malachite green phosphate assay kit (POMG-25H) was purchased from BioAssay System (Gentaur). Electron microscopy reagents were purchased from Electron Microscopy Sciences. Media components were purchased from BD Biosciences. Doxycycline hydrochloride was purchased from Biochem pharmaceutical.

### Generation of *glmU* conditional gene mutant in *M*. *tuberculosis*

The hexa-His tag in the pST-KiT construct[15] was replaced with an N-terminal FLAG tag, and the tetracycline repressor gene (*tetR*) was replaced with a reverse tetR (*r-tetR*) from pTC28S15-OX [64] to create plasmid pST-KirT. To generate the integrating shuttle plasmid pST-KirT-*glmU*_*Mtb*_, the *glmU*_*Mtb*_ gene was excised from pQE2-*glmU*_*Mtb*_ using *NdeI-HindIII* digestion and was subcloned into the corresponding sites on pST-KirT. The resulting pST-KirT-*glmU* construct expresses GlmU_Mtb_ in the absence of inducer ATc. Upon addition of ATc, ATc binds to the r-TetR repressor resulting in the conformational change that would allow it to bind to the operator seqeunces in P_myc_tetO (S1 Fig) [64]. The integration-proficient plasmid containing the inducible *glmU*_*Mtb*_ gene was electroporated into mycobacterial cells to create the merodiploid strain *Rv*::*glmU*_*Mtb*_. 5’ and 3’ genomic flank sequences of *glmU*_*Mtb*_ (approximately 1 kb on either side) were amplified, the amplicons digested with *PflMI*, and ligated with the antibiotic resistance cassette along with the *oriE* and *cosλ* fragments generated from pYUB1474 construct, to generate the allelic exchange substrate (AES) [65]. The AES was linearized using the unique *PacI* site and then cloned into temperature sensitive shuttle phagemid phAE159 at the *PacI* site. A conditional gene replacement mutant of *RvΔglmU* was created from the merodiploids with the help of specialized transduction methodology (S1A Fig) [66]. *RvΔglmU* recombinants obtained were analyzed by PCR amplification to verify the fidelity of the recombination event.

### Analysis of growth patterns

H37Rv (*Rv*) and *RvΔglmU* cultures were grown in Middlebrook 7H9 medium supplemented with 10% ADC (albumin, dextrose and catalase), or in 7H11 medium supplemented with 10% OADC (oleic acid, ADC). To analyze bacterial growth *in vitro*, *Rv* and *Rv*∆*glmU* mutant bacterial cultures were inoculated at A_600_ of 0.1, in the presence or absence of anhydrotetracycline (ATc), and A_600_ was measured every 24 h for 6 or 8 days. For spotting analysis, cells were harvested by centrifugation, washed twice with PBST (0.05% Tween 80) to remove ATc, resuspended in 7H9 medium, and serially diluted in the same medium, followed by spotting 10 μl aliquots of the various cell dilutions on 7H11 agar plates to assess cell viability. To determine the impact of GlmU_Mtb_ depletion during hypoxia in *Rv* and *RvΔglmU* strains, we established hypoxia in 1.5 ml HPLC tubes or 500 ml flasks with penetrable caps, following modified Wayne model [35]. ATc (2 μg/ml) or isoniazid (INH) (50 ng/ml) were injected into the cultures at different time points and the number of CFUs were determined after 42 days. Scanning and transmission electron microscopy (SEM & TEM) analysis of *Rv* and *Rv*∆*glmU* mutant grown in the presence or absence of ATc were performed as described earlier [67]. Transmission electron microscopy was performed using standard protocols. Briefly, bacteria was fixed in 2.5% gluteraldehyde and 4% paraformaldehyde, dehydrated in graded series of alcohol and embedded in Epon 812 resin. Ultrathin sections were cut and stained with uranyl acetate and lead citrate [68]. SEM images were procured using Carl Zeiss Evo LS scanning electron microscope, and TEM images were captured using Tecnai G2 20 twin (FEI) transmission electron microscope.

### Generation of *glmU*_*Mtb*_ mutant constructs and western blotting analysis

Site directed mutations of *glmU*_*Mtb*_ were generated with the help of overlapping PCR and the amplicons were cloned into *NdeI-HindIII* sites of pQE-2, pNit and pST-KT vectors [15,69]. The tetracylin repressor (TetR) expressed from the plasmids binds to the operator sequence in the promoter P_myc_tetO in the absence of ATc (S1B Fig) [70]. Addition of ATc to TetR alleviates the repression thus inducing the expression of GlmU. pST-KT-*glmU* was electroporated into *Rv* to generate *Rv*::*glmU*_*tet-on*_ strain. pNit-*glmU* (wild type and mutated) constructs were electroporated into *Rv*∆*glmU* to generate *Rv*∆*glmU*::*glmU*_*wt/mutant*_ strains. *Rv* and *Rv*∆*glmU*::*glmU*_*wt/mutant*_ strains were grown in the presence or absence of ATc as described above. GlmU_Mtb_ was expressed and purified using plasmid pQE2-GlmU_Mtb_, as described earlier [15]. Whole cell lysates (WCL) isolated from *Rv*, *Rv*∆*glmU* and *Rv*∆*glmU*::*glmU*_*wt/mutant*_ strains that had been grown for 5 days in presence or absence of Atc, were resolved on 10% SDS-PAGE, transferred to nitrocellulose membrane, and probed with anti-GlmU_Mtb_ and anti-GroEL1 antibodies as described earlier [15,67].

### *Ex vivo* and *in vivo* infections

THP1 infection experiments were carried out with either unlabelled or FITC-labelled *Rv* and *Rv*∆*glmU* strains at 1:10 MOI, as described earlier [71]. For examination of cells under a fluorescence microscope, infected cells (48 h post-infection) were labelled with Lyso Tracker red DND 99 dye (50 nM) and mounted with Antifade (Invitrogen) mounting agent. To determine CFUs per infected cell, the infected cells were lysed in PBS containing 0.1% TritonX-100 for 15 min and different dilutions were plated on OADC-containing 7H11 agar plates. For animal infection experiments, *Rv* and *Rv*∆*glmU* strains grown till A_600_ of 0.6 were used to infect 3 to 4 week old guinea pigs or ~ 2 month old mice as described previously [72,73]. We initially used guinea pig model system as it has robust immune response. However, for the remaining experiments we chose to use Balb/C mice model of infection as the cost associated with performing the experiments and the amount of Oxa33 required for guinea pig experiments was prohibitive. To determine the implantation dosage, the bacillary load in the lungs of guinea pigs or mice was determined 24 h post-infection. To investigate the impact of *glmU*_*Mtb*_ depletion on survival of the pathogen, doxycycline hydrochloride (Dox, 1 mg/ kg with 5% dextrose in drinking water) was provided to *Rv* and *Rv*∆*glmU*-infected animals as indicated, either from the time of the infection (guinea pig experiment), or 4 weeks post-infection (mice infection experiments). To assess the impact of INH or Oxa33 treatment on pathogen survival, *Rv*-infected mice (4 weeks post-infection) were supplied with INH (25 mg/ kg body weight, with 5% dextrose in drinking water) or Oxa33 (50 mg/ kg body weight, with 2.5% Tween 80, injected intra peritoneally) every third day for 8 weeks. Bacillary loads in the lungs and spleens of infected guinea pigs and mice were determined 4 weeks and 12 weeks post-infection. Histopathological evaluation of the harvested organs was performed as described earlier [67,72,73].

### Shape based screening and molecular docking studies

ROCS (Rapid Overlay of Chemical Structures), a shape based technique for rapid similarity analysis was used to assess the compounds. Gaussians and shape tanimoto were used to assess the volume and shape overlaps of the compounds, respectively. As the chemical functionality is critical, the chemical feature based similarity was also considered using ROCS colour score whose force field was composed of SMARTS patterns of the chemical functions [74,75]. The shape tanimoto score and scaled color score were considered during the selection of the compounds for further virtual screening. The compounds selected were subjected to molecular docking studies using Glide *v*5.8 of Schrödinger molecular modelling suite 2012 (Glide *v*5.8, Schrödinger). The compounds were subjected to a series of docking protocols–high throughput virtual screening (HTVS), standard precision (SP) and extra precision (XP) docking. As the docking progresses from HTVS to XP, the algorithm differs, which starts from a simple docking of compounds and ends with docking protocol with high precision and parameterization while cutting off the number of compounds.

### Synthesis of 4-(4-(benzyloxy)benzylidene)-2-(naphthalen-1-yl)oxazol-5(4H)-one

To the glycine solution (3.0 g, 39.89 mmol) in water under constant stirring at 0°C, NaOH (3.19 g, 79.78 mmol) was added. This was followed few minutes later by the addition of 1-naphthoyl chloride (7.20 mL, 47.86 mmol) in 1, 4-Dioxane (20 ml) and the contents were stirred at room temperature for 6 h. The reaction mixture was concentrated to half the volume and 60 ml EtOAc was added. The EtOAc layer was washed with sat NaHCO_3_ (2 × 30 mL) followed by H_2_O (2 × 20 mL). The separated organic layer was dried and concentrated over anhydro Na_2_SO_4_ to obtain solid compound, which was washed with hexanes to get 2-(1-naphthamido) acetic acid (8.30 g, 90%) as a white solid (M.P. 152°C^1^). 2-(1-naphthamido)acetic acid (2.0 g, 8.73 mmol), NaOAc (0.21 g, 2.62 mmol) and 4-benzyloxybenzaldehyde (1.85 g, 8.73 mmol) were taken in acetic anhydride and heated at 106°C for 3 h. The solid formed were filtered and washed with water to remove traces of acetic anhydride, and ethanol to remove unreacted aldehyde and other organic impurities. Final compound 4-(4-(benzyloxy)benzylidene)-2-(naphthalen-1-yl)oxazol-5(4H)-one (Oxa33; 3.14 g, 88%), purified as a yellow solid, was confirmed with nuclear magnetic resonance (NMR) [76].

### Isothermal titration calorimetry

To investigate the binding of Oxa33 to GlmU_Mtb_, we performed Isothermal Titration Calorimetry (MicroCal 2000 VP-ITC, GE Healthcare) [28]. Oxa33 was re-suspended in dialysis buffer (25 mM Tris pH 7.4, NaCl 140 mM and 15% glycerol), 100 μM of MgCl_2_ containing 2% DMSO. 625 μM of Oxa33 was injected for titrations from syringe (rotating at 307 rpm) into ITC cell containing 25 μM of GlmU or blank buffer at 25°C. Each injection lasted for 20 sec with 300 sec interval between every step. The quantity of heat associated by every injection was calculated by combining the area beneath every heat burst curve (microcalories/second vs. seconds). Data was corrected for the buffer signal and fitting was done by one-site binding model. Origin software (version 7.0) was used to obtain different thermodynamic binding parameters.

### *In vitro* cytotoxicity

Oxa33 was evaluated for its cytotoxic activity in THP1 cells with the help of alamar blue assay. Serially diluted inhibitors (in 2.5% DMSO) incubated with 5 x 10^3^ differentiated THP-1 cells in 96 well plates for 3 days. After 3 days cells were incubated for 5 h with 10 μl of alamar blue and color development was measured using micro-plate reader at 570 nm.

### Docking and molecular dynamics simulations studies of GlmU_Mtb_ with Oxa33

Molecular dynamics (MD) simulation for the protein-ligand complex was carried out for a time scale of 20 ns so as to analyze the stability of molecular interactions between ligand and protein employing Newton’s Laws of Motions. Desmond molecular dynamics system *v*3.1 was used for carrying out the simulations employing OPLS-AA force field [77]. The protein-ligand complex was solvated using TIP3P water model which was setup as an orthorhombic solvent box, keeping a cut-off of 10 Å from any solute atom in all directions [78]. Na^+^ counter ions were added in order to neutralize the system. A cut-off of 14 Å was maintained for calculating the solvent-solvent and solute-solvent non-bonded interactions. Initially, the system was minimized keeping the convergence threshold criteria of 1.0 kcal.mol^-1^.Å^-1^ so as to allow the adjustment of atoms to the system environment. A simulation for each system was performed using isothermal-isobaric ensemble (NPT) including a relaxation process. Under NPT, the system was simulated for 12 ps using a Berendsen thermostat and a Berendsen barostat with temperature of 10K and a pressure of 1 atm. The later step of relaxation protocol included the simulation of the system for 24 ps with a temperature of 300 K and 1 atm pressure with and without restraints on solute heavy atoms. M-SHAKE algorithm was used with an integration time step of 2 fs for rearranging the hydrogen bonds in the simulation [79]. The temperature and pressure of the system were maintained at 300 K and 1.013 atm respectively. The molecular dynamics simulation was run for 20 ns recording the trajectory frames at an interval of every 4.8 ps and the trajectory analysis was carried out using the Simulation Event Analysis of Desmond.

### Determination of percentage inhibition, IC_50_ and MIC

Uridyltranferase assays were performed using malachite green phosphate detection kit as described previously [17]. Acetyltransferase activity of GlmU_Mtb_ was carried out in the presence of 500 μM each of GlcN-1-P and acetyl-CoA in a 30 μl reaction volume for 30 min at 30°C as described earlier [80]. To determine the percent inhibition by different compounds the enzyme was preincubated with either 5% DMSO or 100 μM compounds for 30 min prior to performing uridyltransferase activity assays. In order to determine the IC_50_ values, GlmU_wt/mutant_ proteins were preincubated with different concentrations of Oxa33 compound for 30 min followed by the uridyltransferase assay. To determine minimum inhibitory concentration (MIC), 5x10^5^ bacteria of *Rv* or *Rv*::*glmU*_*tet-on*_ (overexpressing GlmU_Mtb_) cultures (grown in the presence or absence of 2 μg/ml ATc) were mixed with 100 μl of 2.5% DMSO or different concentrations of Oxa33/ INH in 96-well plates, and incubated at 37°C for 6 days. After 6 days, 40 μl of resazurin dye (0.02% in 5% Tween-80) was added to each well and the colour change was observed after 12 h.

### Ethical statement

Experimental protocol for the animal experiments was approved by the Institutional Animal Ethics Committee of National Institute of Immunology, New Delhi, India (the approval number is IAEC# 315/13). The approval is as per the guidelines issued by Committee for the Purpose of Control and Supervision of Experiments on Animals (CPCSEA), Govt. of India.

### Statistical analysis

Student’s *t*-test (two tailed non parametric) was used to analyze the significance of cell wall thickness, THP1 and animal infection experimental results. SigmaPlot version 10.0 and GraphPad Prism version 5.0 was used for the statistical analysis and for plotting the results.

## Supporting Information

S1 Text Supplementary Methods and Results. Designing and development of allosteric site inhibitors of GlmU_Mtb_. Screening of inhibitors. Isothermal Titration Calorimetry. Docking and molecular dynamics simulations. H-bond analysis. Survival curve and maximum dose tolerance. Estimation of Oxa33 from treated mice lungs.
(DOC)

S1 Fig Schematic representating regulation of Tet-off (A) and Tet-on systems (B). (TIF)

S2 Fig Effect of GlmU_Mtb_ depletion on spleen during guinea pig infection. Guinea pigs (six per group) were infected with *Rv* or *Rv*∆*glmU* and Dox was provided in the water as indicated in Fig 5. **(A)** Overall pathology of the infected spleens from the guinea pigs 4 weeks post infection. **(B)** CFU data from guinea pig spleens. ***p<0.0001 or, two tailed non parametric *t*-test, mean, error bars indicate s.e.m.
(TIF)

S3 Fig Depletion of GlmU_Mtb_ from infected spleen or any stage of growth leads to clearence of pathogen. **(A)** *Rv* and *Rv*∆*glmU* cultures were inoculated at an initial *A*_600_ of 0.1. ATc was added to the *Rv* culture on day 1 (a) and *Rv*∆*glmU* cultures were either grown in the absence of ATc or was supplemented with ATc in the growth media on 1^st^ (a), 3^rd^ (b) or 5^th^ (c) day. Serially diluted cultures were spotted on 7H11 agar plates after day 1, 3, 5 and 7. The experiment was performed in triplicates. **(B)** BALB/c mice (6 to 9 / group) were infected with *Rv* and *Rv*∆*glmU* strains. 28 days post infection Dox was provided for *Rv* and *Rv*∆*glmU* infected mice and one group of *Rv*∆*glmU* infected mice were administered with the vehicle control for the next 56 days. CFUs were enmurated from the spleens of infected mice on day 28 and day 84 post infection. At 28 days post infection mean CFUs for the spleens of *Rv* and *Rv*∆*glmU* infected mice were 2.9 and 3.15 on log_10_ scale and 84 days post infection mean CFUs for *Rv* +Dox or *Rv*∆*glmU* +Dox and *Rv*∆*glmU*–Dox infected spleens were 3.67, 0 and 3.5 on log_10_ scale. ***p<0.0005, two tailed non parametric *t*-test, error bars indicate s.e.m.
(TIF)

S4 Fig Structural comparision of *H*. *influenzae* and *Mtb* allosteric site. **(A)** Surface representation of GlmU_HI_ wherein its allosteric residues are highlighted. The allosteric site of GlmU_HI_, comprises of highly lipophilic surface and a distal depression surface. **(B)** Interction of GlmU_HI_ allosteric inhibitor at the allosteric site. Hydrophobic residues are coloured in green, polar residues in blue, negatively charged residues in red. Hydrogen bond interaction of the ligand with Gln231 is shown in pink color; Glu224 is also involved in the hydrogen bonding which is not represented here. Leu133, Tyr139, Met221, Leu235 were found to be involved in strong hydrophobic interactions with the inhibitor. Interactions were plotted with LIGPLOT. **(C)** Sequence alignment of GlmU_HI_ and GlmU_Mtb_ allosteric site residues. Conserved residues are highlighted in red boxes. **(D)** Table displaying allosteric residues of GlmU_HI_ and GlmU_Mtb_. Some of the critical residues such as Tyr150, Gln243 and Leu247 of GlmU_Mtb_ are seen to be conserved.
(TIF)

S5 Fig Design, development and synthesis of anti-GlmU_Mtb_ inhibitor. **(A)** Virtual screening workflow used towards the identification of allosteric inhibitors for GlmU_Mtb_. Shape model of allosteric inhibitor of GlmU_HI_ was generated using ROCS software. In the initial set of screening, a total of 43 compounds were identified and tested for their ability to inhibit uridyltransferase reaction. 10 among the 43 compounds showed ~90% inhibition at 100 μM. Of these, an oxozolone derivative was identified and further chemically derivatized to a library of 52 compounds. One compound from these, Oxa33, which showed ~90% inhibition was considered for further studies. **(B)** Nuclear magnetic resonance spectra of purified Oxa33 (Yellow solid). ^1^H NMR (400 MHz, CDCl_3_): δ 9.51 (d, *J* = 8.8 Hz, 1H), 8.38 (d, *J* = 8.8 Hz, 1H), 8.25 (d, *J* = 8.4 Hz, 2H), 8.10 (d, *J* = 8.4 Hz, 1H), 7.93 (d, *J* = 8.0 Hz, 1H), 7.74 (t, *J* = 8.0 Hz, 1H), 7.68–7.56 (m, 2H), 7.49–7.33 (m, 5H), 7.29 (s, 1H), 7.12 (d, *J* = 8.4 Hz, 2H), 5.15 (s, 2H). Anal calcd for: C_27_H_19_NO_3_: C, 79.98; H, 4.72; N, 3.45% Found C, 79.92; H, 4.83; N, 3.54%.
(TIF)

S6 Fig ITC, MD simulation and DSF studies of Oxa33. **(A)** Isothermal titration calorimetry results of Oxa33 with GlmU_Mtb_. Released heat increased over the period of time (*μ*cal/sec) is presented in upper panel while corresponding binding isotherm (fitted for one site) presented in lower panel. **(B)** rmsd plot for GlmU_Mtb_-oxa33 complex simulated for a period of 20ns. The complex was found to be unstable during the initial time period which can be owed for its relaxation. After 8 ns, the complex was observed to be stable indicating the strong binding affinity of Oxa33 towards GlmU_Mtb_ **(C)** Hydrogen bond analysis of Oxa33 analyzed during the simulation time period. **(D)** Purified GlmU_Mtb_ mutants (single band) specified by the docking studies of Oxa33. **(E)** Differential Scanning Fluorimetry (DSF; performed in triplicates) results of Oxa33 with GlmU_Mtb_. Left panel representing melting curve of GlmU_Mtb_ in presence of 5% DMSO. While right graph is showing -3°C T_m_ shift upon Oxa33 incubation with GlmU_Mtb_ protein.
(TIF)

S7 Fig GlmU_Mtb_ overexpression, cytotoxicity and effect of INH upon Rv or overexpressing strains. **(A)** *Rv* and *Rv*::*glmU*_*tet-on*_ strains were inoculated at an initial *A*_600_ of 0.1 in the presence of ATc (2 μg/ml) and grown up to 5 days. WCLs were resolved and probed with anti-GlmU and anti-GroEL1 antibodies. **(B)** *Rv* culture was treated with sub lethal dose of Oxa33 (40 μM) and variable concentrations of INH for 6 days followed by resazurine addition. Results show decrease in MICs of INH from 32 ng/ml to 16 ng/ml. The experiment was pefromed in triplicates. **(C)** Bar graph presents the cytoxicity of the Oxa33 on THP1 cells during the 3 days of treatment. Cell viability was checked with alamar blue based assay and absorbance at 570 nm was plotted with increasing concentrations of Oxa33 inhibitor. The experiment was performed in triplicates and the error bars represent s.e.m. **(D)** CFUs count results of THP1 cells infected with *Rv* (for 24 h post infection) followed by treatment with various concentrations of INH (in water) for 3 days. CFU (log_10_) per well shows gradual increase in bacterial death with increasing INH doses. **p<0.005, two tailed non parametric *t*-test, mean, s.e.m., n = 3). **(E)** Graph illustrate CFU numbers from fully infected THP1 cells (24 h) with *Rv*:: *glmU*_*tet-on*_. Abundant GlmU_Mtb_ expression in *Rv*::*glmU*_*tet-on*_ infected THP1 cells (with ATc) does not affect INH efficacy, which gives similar results as *Rv*. **p<0.005, two tailed non parametric *t*-test,. error bars represent s.e.m.
(TIF)

S8 Fig Dose tolerance and mice survival curves. **(A)** Maximum dose tolerance graph showing relative change in body weight of mice (4 mice / group) during the course of Oxa33 administration for 30 days. **(B)** Survival curve of mice (4 mice / group) treated with different concentrations of Oxa33 for 30 days.
(TIF)

S9 Fig *In-vivo* efficacy on *Mtb* load in infected mice spleen and toxicity of Oxa33. **(A)** *Mtb* bacterial load in the spleen of totally infected mice (7 to 11 mice /group) (for 28 days) and treated with Oxa33 or INH for subsequently 56 days. ***p<0.0007 or **p<0.002, two tailed non parametric *t*-test, the error bars represent s.e.m. **(B)** High power (400x) photomicrograph of hematoxylin and eosin stained spleen, brain, heart, liver and lungs of untreated mice or mice treated with Oxa33 for 8 weeks. Spleen is showing usual parenchyma. WP = White Pulp, RP = Red Pulp. While, section from hippocampal area of brain showing several degenerated neuron in the neuronal layer. Section of heart is showing normal cardiac muscle fibres. Photomicrograph of liver depicts typical hepatic parenchyma. PV = Portal Vein, BD = Bile Duct. Lung section also represents usual lung parenchyma. BL = Bronchial Lumen, AS = Alveolar space.
(TIF)

S10 Fig Standard curve for Oxa33 and its estimation in treated mice lungs. **(A)** Absorbance spectra of Oxa33 resuspended in tetrahydrofuran (THF). It showed a peak at 401 nm. **(B)** Absorbance readings (401 nm; performed in triplicates; A, B and C) of Oxa33 at different concentrations **(C)** Standard curve of Oxa33 showing linear regression with a slope of 0.0497 OD / μg. **(D)** Table showing Oxa33 concentrations (μg/lung) in the lungs of mice treated with 50 mg /kg (body weight) for 3 or 8 weeks.
(TIF)

S11 Fig Model for the UDP-GlcNAc synthesis pathways in *Mtb*. Model shows De novo pathway for UDP-GlcNAc synthesis is mediate by GlmS, GlmM and GlmU enzymes. Shaded pathway is conserved in *Mtb*. UPD-GlcNAc can inhibit uridyltransferase activity by feedback inhibition mechanism. Also GlcNAc from host resources or from cell wall recycling can be transported inside the bacteria and further metabolized and feeded into the de novo pathways through GlmS/ GlmU mediated reactions. Question marks show that these pathways are still not characterized in *Mtb*. Dashed lines shows possible input of substrates or unknown pathway while complete lines shows established and known pathways for UDP-GlcNAc synthesis.
(TIF)

S1 Table List of bacterial strains, plasmids and phages used in the study. (DOC)

## References

[1] Smith I (2003) Mycobacterium tuberculosis pathogenesis and molecular determinants of virulence. Clin Microbiol Rev 16: 463–496. 12857778 10.1128/CMR.16.3.463-496.2003 PMC164219

[2] McNeil MR, Brennan PJ (1991) Structure, function and biogenesis of the cell envelope of mycobacteria in relation to bacterial physiology, pathogenesis and drug resistance; some thoughts and possibilities arising from recent structural information. Res Microbiol 142: 451–463. 1871433 10.1016/0923-2508(91)90120-y

[3] Brennan PJ, Nikaido H (1995) The envelope of mycobacteria. Annu Rev Biochem 64: 29–63. 7574484 10.1146/annurev.bi.64.070195.000333

[4] Lee A, Wu SW, Scherman MS, Torrelles JB, Chatterjee D, et al (2006) Sequencing of oligoarabinosyl units released from mycobacterial arabinogalactan by endogenous arabinanase: identification of distinctive and novel structural motifs. Biochemistry 45: 15817–15828. 17176104 10.1021/bi060688d PMC2532846

[5] Zumla A, Nahid P, Cole ST (2013) Advances in the development of new tuberculosis drugs and treatment regimens. Nat Rev Drug Discov 12: 388–404. 10.1038/nrd4001 23629506

[6] Mengin-Lecreulx D, van Heijenoort J (1994) Copurification of glucosamine-1-phosphate acetyltransferase and N-acetylglucosamine-1-phosphate uridyltransferase activities of Escherichia coli: characterization of the glmU gene product as a bifunctional enzyme catalyzing two subsequent steps in the pathway for UDP-N-acetylglucosamine synthesis. J Bacteriol 176: 5788–5795. 8083170 10.1128/jb.176.18.5788-5795.1994 PMC196783

[7] Gehring AM, Lees WJ, Mindiola DJ, Walsh CT, Brown ED (1996) Acetyltransfer precedes uridylyltransfer in the formation of UDP-N-acetylglucosamine in separable active sites of the bifunctional GlmU protein of Escherichia coli. Biochemistry 35: 579–585. 8555230 10.1021/bi952275a

[8] Crick DC, Mahapatra S, Brennan PJ (2001) Biosynthesis of the arabinogalactan-peptidoglycan complex of Mycobacterium tuberculosis. Glycobiology 11: 107R–118R. 11555614 10.1093/glycob/11.9.107r

[9] Alderwick LJ, Birch HL, Mishra AK, Eggeling L, Besra GS (2007) Structure, function and biosynthesis of the Mycobacterium tuberculosis cell wall: arabinogalactan and lipoarabinomannan assembly with a view to discovering new drug targets. Biochem Soc Trans 35: 1325–1328. 17956343 10.1042/BST0351325

[10] Barreteau H, Kovac A, Boniface A, Sova M, Gobec S, et al (2008) Cytoplasmic steps of peptidoglycan biosynthesis. Fems Microbiology Reviews 32: 168–207. 10.1111/j.1574-6976.2008.00104.x 18266853

[11] Birch HL, Alderwick LJ, Bhatt A, Rittmann D, Krumbach K, et al (2008) Biosynthesis of mycobacterial arabinogalactan: identification of a novel alpha(1—>3) arabinofuranosyltransferase. Mol Microbiol 69: 1191–1206. 10.1111/j.1365-2958.2008.06354.x 18627460 PMC2610374

[12] Mills JA, Motichka K, Jucker M, Wu HP, Uhlik BC, et al (2004) Inactivation of the mycobacterial rhamnosyltransferase, which is needed for the formation of the arabinogalactan-peptidoglycan linker, leads to irreversible loss of viability. J Biol Chem 279: 43540–43546. 15294902 10.1074/jbc.M407782200

[13] Dover LG, Cerdeno-Tarraga AM, Pallen MJ, Parkhill J, Besra GS (2004) Comparative cell wall core biosynthesis in the mycolated pathogens, Mycobacterium tuberculosis and Corynebacterium diphtheriae. FEMS Microbiol Rev 28: 225–250. 15109786 10.1016/j.femsre.2003.10.001

[14] Vilcheze C, Av-Gay Y, Attarian R, Liu Z, Hazbon MH, et al (2008) Mycothiol biosynthesis is essential for ethionamide susceptibility in Mycobacterium tuberculosis. Mol Microbiol 69: 1316–1329. 10.1111/j.1365-2958.2008.06365.x 18651841 PMC2628429

[15] Parikh A, Kumar D, Chawla Y, Kurthkoti K, Khan S, et al (2013) Development of a new generation of vectors for gene expression, gene replacement, and protein-protein interaction studies in mycobacteria. Appl Environ Microbiol 79: 1718–1729. 10.1128/AEM.03695-12 23315736 PMC3591980

[16] Verma SK, Jaiswal M, Kumar N, Parikh A, Nandicoori VK, et al (2009) Structure of N-acetylglucosamine-1-phosphate uridyltransferase (GlmU) from Mycobacterium tuberculosis in a cubic space group. Acta Crystallogr Sect F Struct Biol Cryst Commun 65: 435–439. 10.1107/S1744309109010252 19407371 PMC2675579

[17] Jagtap PK, Soni V, Vithani N, Jhingan GD, Bais VS, et al (2012) Substrate-bound crystal structures reveal features unique to Mycobacterium tuberculosis N-acetyl-glucosamine 1-phosphate uridyltransferase and a catalytic mechanism for acetyl transfer. J Biol Chem 287: 39524–39537. 10.1074/jbc.M112.390765 22969087 PMC3501063

[18] Sassetti CM, Boyd DH, Rubin EJ (2003) Genes required for mycobacterial growth defined by high density mutagenesis. Mol Microbiol 48: 77–84. 12657046 10.1046/j.1365-2958.2003.03425.x

[19] Zhang YJ, Ioerger TR, Huttenhower C, Long JE, Sassetti CM, et al (2012) Global Assessment of Genomic Regions Required for Growth in Mycobacterium tuberculosis. PLoS Pathog 8: e1002946 10.1371/journal.ppat.1002946 23028335 PMC3460630

[20] Zhang W, Jones VC, Scherman MS, Mahapatra S, Crick D, et al (2008) Expression, essentiality, and a microtiter plate assay for mycobacterial GlmU, the bifunctional glucosamine-1-phosphate acetyltransferase and N-acetylglucosamine-1-phosphate uridyltransferase. Int J Biochem Cell Biol 40: 2560–2571. 10.1016/j.biocel.2008.05.003 18573680 PMC2602953

[21] Jackson M, McNeil MR, Brennan PJ (2013) Progress in targeting cell envelope biogenesis in Mycobacterium tuberculosis. Future Microbiol 8: 855–875. 10.2217/fmb.13.52 23841633 PMC3867987

[22] Moraes GL, Gomes GC, Monteiro de Sousa PR, Alves CN, Govender T, et al (2015) Structural and functional features of enzymes of Mycobacterium tuberculosis peptidoglycan biosynthesis as targets for drug development. Tuberculosis (Edinb) 95: 95–111. 25701501 10.1016/j.tube.2015.01.006 PMC4659487

[23] Urbaniak MD, Collie IT, Fang W, Aristotelous T, Eskilsson S, et al (2013) A novel allosteric inhibitor of the uridine diphosphate N-acetylglucosamine pyrophosphorylase from Trypanosoma brucei. ACS Chem Biol 8: 1981–1987. 10.1021/cb400411x 23834437 PMC3780468

[24] Moir DT, Di M, Moore RA, Schweizer HP, Woods DE (2008) Cellular reporter screens for inhibitors of Burkholderia pseudomallei targets in Pseudomonas aeruginosa. Trans R Soc Trop Med Hyg 102 Suppl 1: S152–162. 10.1016/S0035-9203(08)70033-6 19121678 PMC2709407

[25] Pereira MP, Blanchard JE, Murphy C, Roderick SL, Brown ED (2009) High-throughput screening identifies novel inhibitors of the acetyltransferase activity of Escherichia coli GlmU. Antimicrob Agents Chemother 53: 2306–2311. 10.1128/AAC.01572-08 19349513 PMC2687215

[26] Doig P, Boriack-Sjodin PA, Dumas J, Hu J, Itoh K, et al (2014) Rational design of inhibitors of the bacterial cell wall synthetic enzyme GlmU using virtual screening and lead-hopping. Bioorg Med Chem 22: 6256–6269. 10.1016/j.bmc.2014.08.017 25262942

[27] Mochalkin I, Lightle S, Narasimhan L, Bornemeier D, Melnick M, et al (2008) Structure of a small-molecule inhibitor complexed with GlmU from Haemophilus influenzae reveals an allosteric binding site. Protein Sci 17: 577–582. 10.1110/ps.073271408 18218712 PMC2248321

[28] Buurman ET, Andrews B, Gao N, Hu J, Keating TA, et al (2011) In vitro validation of acetyltransferase activity of GlmU as an antibacterial target in Haemophilus influenzae. J Biol Chem 286: 40734–40742. 10.1074/jbc.M111.274068 21984832 PMC3220512

[29] Larsen NA, Nash TJ, Morningstar M, Shapiro AB, Joubran C, et al (2012) An aminoquinazoline inhibitor of the essential bacterial cell wall synthetic enzyme GlmU has a unique non-protein-kinase-like binding mode. Biochem J 446: 405–413. 10.1042/BJ20120596 22721802

[30] Min J, Lin D, Zhang Q, Zhang J, Yu Z (2012) Structure-based virtual screening of novel inhibitors of the uridyltransferase activity of Xanthomonas oryzae pv. oryzae GlmU. Eur J Med Chem 53: 150–158. 10.1016/j.ejmech.2012.03.051 22521370

[31] Singh VK, Das K, Seshadri K (2012) Kinetic modelling of GlmU reactions—prioritization of reaction for therapeutic application. PLoS One 7: e43969 10.1371/journal.pone.0043969 22952829 PMC3428340

[32] Li Y, Zhou Y, Ma Y, Li X (2011) Design and synthesis of novel cell wall inhibitors of Mycobacterium tuberculosis GlmM and GlmU. Carbohydr Res 346: 1714–1720. 10.1016/j.carres.2011.05.024 21704310

[33] Tran AT, Wen D, West NP, Baker EN, Britton WJ, et al (2013) Inhibition studies on Mycobacterium tuberculosis N-acetylglucosamine-1-phosphate uridyltransferase (GlmU). Org Biomol Chem 11: 8113–8126. 10.1039/c3ob41896k 24158720

[34] Chao MC, Rubin EJ (2010) Letting sleeping dos lie: does dormancy play a role in tuberculosis? Annu Rev Microbiol 64: 293–311. 10.1146/annurev.micro.112408.134043 20825351

[35] Wayne LG, Hayes LG (1996) An in vitro model for sequential study of shiftdown of Mycobacterium tuberculosis through two stages of nonreplicating persistence. Infect Immun 64: 2062–2069. 8675308 10.1128/iai.64.6.2062-2069.1996 PMC174037

[36] Blokpoel MC, Murphy HN, O'Toole R, Wiles S, Runn ES, et al (2005) Tetracycline-inducible gene regulation in mycobacteria. Nucleic Acids Res 33: e22 15687380 10.1093/nar/gni023 PMC548381

[37] Karakousis PC, Williams EP, Bishai WR (2008) Altered expression of isoniazid-regulated genes in drug-treated dormant Mycobacterium tuberculosis. J Antimicrob Chemother 61: 323–331. 18156607 10.1093/jac/dkm485

[38] Parikh A, Verma SK, Khan S, Prakash B, Nandicoori VK (2009) PknB-mediated phosphorylation of a novel substrate, N-acetylglucosamine-1-phosphate uridyltransferase, modulates its acetyltransferase activity. J Mol Biol 386: 451–464. 10.1016/j.jmb.2008.12.031 19121323

[39] Alavi HA, Moscovic EA (1996) Immunolocalization of cell-wall-deficient forms of Mycobacterium tuberculosis complex in sarcoidosis and in sinus histiocytosis of lymph nodes draining carcinoma. Histol Histopathol 11: 683–694. 8839759

[40] BERAN MH V., KAUSTOVA J., DVORSKA L., PAVLIK I. (2006) Cell wall deficient forms of mycobacteria: a review. Veterinarni Medicina 51: 365–389.

[41] Sonnenburg JL, Xu J, Leip DD, Chen CH, Westover BP, et al (2005) Glycan foraging in vivo by an intestine-adapted bacterial symbiont. Science 307: 1955–1959. 15790854 10.1126/science.1109051

[42] Renzi F, Manfredi P, Dol M, Fu J, Vincent S, et al (2015) Glycan-foraging systems reveal the adaptation of Capnocytophaga canimorsus to the dog mouth. MBio 6: e02507 10.1128/mBio.02507-14 25736888 PMC4358021

[43] Renzi F, Manfredi P, Mally M, Moes S, Jeno P, et al (2011) The N-glycan glycoprotein deglycosylation complex (Gpd) from Capnocytophaga canimorsus deglycosylates human IgG. PLoS Pathog 7: e1002118 10.1371/journal.ppat.1002118 21738475 PMC3128124

[44] Gisin J, Schneider A, Nagele B, Borisova M, Mayer C (2013) A cell wall recycling shortcut that bypasses peptidoglycan de novo biosynthesis. Nat Chem Biol 9: 491–493. 10.1038/nchembio.1289 23831760

[45] Engels FK, Mathot RA, Verweij J (2007) Alternative drug formulations of docetaxel: a review. Anticancer Drugs 18: 95–103. 17159596 10.1097/CAD.0b013e3280113338

[46] Coors EA, Seybold H, Merk HF, Mahler V (2005) Polysorbate 80 in medical products and nonimmunologic anaphylactoid reactions. Ann Allergy Asthma Immunol 95: 593–599. 16400901 10.1016/S1081-1206(10)61024-1

[47] Lovering AL, Safadi SS, Strynadka NC (2012) Structural perspective of peptidoglycan biosynthesis and assembly. Annu Rev Biochem 81: 451–478. 10.1146/annurev-biochem-061809-112742 22663080

[48] Denholm JT, McBryde ES (2010) The use of anti-tuberculosis therapy for latent TB infection. Infect Drug Resist 3: 63–72. 21694895 10.2147/idr.s8994 PMC3108738

[49] Hart GW, Slawson C, Ramirez-Correa G, Lagerlof O (2011) Cross talk between O-GlcNAcylation and phosphorylation: roles in signaling, transcription, and chronic disease. Annu Rev Biochem 80: 825–858. 10.1146/annurev-biochem-060608-102511 21391816 PMC3294376

[50] Tanner ME (2005) The enzymes of sialic acid biosynthesis. Bioorg Chem 33: 216–228. 15888312 10.1016/j.bioorg.2005.01.005

[51] Itano N, Kimata K (2002) Mammalian hyaluronan synthases. IUBMB Life 54: 195–199. 12512858 10.1080/15216540214929

[52] Swoboda JG, Campbell J, Meredith TC, Walker S (2010) Wall teichoic acid function, biosynthesis, and inhibition. Chembiochem 11: 35–45. 10.1002/cbic.200900557 19899094 PMC2798926

[53] Mikusova K, Mikus M, Besra GS, Hancock I, Brennan PJ (1996) Biosynthesis of the linkage region of the mycobacterial cell wall. J Biol Chem 271: 7820–7828. 8631826 10.1074/jbc.271.13.7820

[54] Skovierova H, Larrouy-Maumus G, Pham H, Belanova M, Barilone N, et al (2010) Biosynthetic origin of the galactosamine substituent of Arabinogalactan in Mycobacterium tuberculosis. J Biol Chem 285: 41348–41355. 10.1074/jbc.M110.188110 21030587 PMC3009860

[55] Wang X, Quinn PJ (2010) Lipopolysaccharide: Biosynthetic pathway and structure modification. Prog Lipid Res 49: 97–107. 10.1016/j.plipres.2009.06.002 19815028

[56] Vimr ER, Kalivoda KA, Deszo EL, Steenbergen SM (2004) Diversity of microbial sialic acid metabolism. Microbiol Mol Biol Rev 68: 132–153. 15007099 10.1128/MMBR.68.1.132-153.2004 PMC362108

[57] Dell A, Galadari A, Sastre F, Hitchen P (2010) Similarities and differences in the glycosylation mechanisms in prokaryotes and eukaryotes. Int J Microbiol 2010: 148178 10.1155/2010/148178 21490701 PMC3068309

[58] Ujita M, Misra AK, McAuliffe J, Hindsgaul O, Fukuda M (2000) Poly-N-acetyllactosamine extension in N-glycans and core 2- and core 4-branched O-glycans is differentially controlled by i-extension enzyme and different members of the beta 1,4-galactosyltransferase gene family. J Biol Chem 275: 15868–15875. 10747980 10.1074/jbc.M001034200

[59] Alderwick LJ, Molle V, Kremer L, Cozzone AJ, Dafforn TR, et al (2006) Molecular structure of EmbR, a response element of Ser/Thr kinase signaling in Mycobacterium tuberculosis. Proc Natl Acad Sci U S A 103: 2558–2563. 16477027 10.1073/pnas.0507766103 PMC1413777

[60] Gill WP, Harik NS, Whiddon MR, Liao RP, Mittler JE, et al (2009) A replication clock for Mycobacterium tuberculosis. Nat Med 15: 211–214. 10.1038/nm.1915 19182798 PMC2779834

[61] Naseem S, Parrino SM, Buenten DM, Konopka JB (2012) Novel roles for GlcNAc in cell signaling. Commun Integr Biol 5: 156–159. 10.4161/cib.19034 22808320 PMC3376051

[62] Handford M, Rodriguez-Furlan C, Orellana A (2006) Nucleotide-sugar transporters: structure, function and roles in vivo. Braz J Med Biol Res 39: 1149–1158. 16981043 10.1590/s0100-879x2006000900002

[63] Konopka JB (2012) N-acetylglucosamine (GlcNAc) functions in cell signaling. Scientifica (Cairo) 2012. 10.6064/2012/489208 PMC3551598 23350039

[64] Klotzsche M, Ehrt S, Schnappinger D (2009) Improved tetracycline repressors for gene silencing in mycobacteria. Nucleic Acids Res 37: 1778–1788. 10.1093/nar/gkp015 19174563 PMC2665214

[65] Jain P, Hsu T, Arai M, Biermann K, Thaler DS, et al (2014) Specialized transduction designed for precise high-throughput unmarked deletions in Mycobacterium tuberculosis. MBio 5: e01245–01214. 10.1128/mBio.01245-14 24895308 PMC4049104

[66] Bardarov S, Bardarov S Jr, Jr., Pavelka MS Jr, Jr., Sambandamurthy V, Larsen M, et al (2002) Specialized transduction: an efficient method for generating marked and unmarked targeted gene disruptions in Mycobacterium tuberculosis, M. bovis BCG and M. smegmatis. Microbiology 148: 3007–3017. 12368434 10.1099/00221287-148-10-3007

[67] Chawla Y, Upadhyay S, Khan S, Nagarajan SN, Forti F, et al (2014) Protein kinase B (PknB) of Mycobacterium tuberculosis is essential for growth of the pathogen in vitro as well as for survival within the host. J Biol Chem 289: 13858–13875. 10.1074/jbc.M114.563536 24706757 PMC4022859

[68] Gupta A, Sharma Y, Dash KN, Verma S, Natarajan VT, et al (2014) Ultrastructural Investigations in an Autosomal Recessively Inherited Case of Dyschromatosis Universalis Hereditaria. Acta Derm Venereol. 10.2340/00015555-2030 25474346

[69] Pandey AK, Raman S, Proff R, Joshi S, Kang CM, et al (2009) Nitrile-inducible gene expression in mycobacteria. Tuberculosis (Edinb) 89: 12–16. 18801704 10.1016/j.tube.2008.07.007 PMC2845969

[70] Ehrt S, Guo XV, Hickey CM, Ryou M, Monteleone M, et al (2005) Controlling gene expression in mycobacteria with anhydrotetracycline and Tet repressor. Nucleic Acids Res 33: e21 15687379 10.1093/nar/gni013 PMC548372

[71] Puri RV, Reddy PV, Tyagi AK (2013) Secreted acid phosphatase (SapM) of Mycobacterium tuberculosis is indispensable for arresting phagosomal maturation and growth of the pathogen in guinea pig tissues. PLoS One 8: e70514 10.1371/journal.pone.0070514 23923000 PMC3724783

[72] Hu Y, Coates AR (2009) Acute and persistent Mycobacterium tuberculosis infections depend on the thiol peroxidase TpX. PLoS One 4: e5150 10.1371/journal.pone.0005150 19340292 PMC2659749

[73] Reddy PV, Puri RV, Chauhan P, Kar R, Rohilla A, et al (2013) Disruption of mycobactin biosynthesis leads to attenuation of Mycobacterium tuberculosis for growth and virulence. J Infect Dis 208: 1255–1265. 10.1093/infdis/jit250 23788726

[74] Kirchmair J, Distinto S, Markt P, Schuster D, Spitzer GM, et al (2009) How to optimize shape-based virtual screening: choosing the right query and including chemical information. J Chem Inf Model 49: 678–692. 10.1021/ci8004226 19434901

[75] Hawkins PC, Skillman AG, Nicholls A (2007) Comparison of shape-matching and docking as virtual screening tools. J Med Chem 50: 74–82. 17201411 10.1021/jm0603365

[76] György Kóczána GCk, Antal Csámpaic, Balogb Erika, Szilvia Bőszea Pál Sohárc, Hudecza Ferenc (2001) Synthesis and characterization of 4-ethoxymethylene-2-[1]-naphthyl-5(4H)-oxazolone and its fluorescent amino acid derivatives Tetrahedron 57: 4589–4598.

[77] William L. Jorgensen DSM, and Julian Tirado-Rives (1996) Development and Testing of the OPLS All-Atom Force Field on Conformational Energetics and Properties of Organic Liquids. Journal of the American Chemical Society 118: 11225–11236.

[78] William L. Jorgensen JC, Jeffry D. Madura, Impey Roger W. and Klein Michael L. (1983) Comparison of simple potential functions for simulating liquid water. The Journal of Chemical Physics 79: 926–935.

[79] Vincent Kräutler WFvGaHH(2001) A fast SHAKE algorithm to solve distance constraint equations for small molecules in molecular dynamics simulations. Journal of Computational Chemistry 22: 501–508.

[80] Zhou Y, Xin Y, Sha S, Ma Y (2011) Kinetic properties of Mycobacterium tuberculosis bifunctional GlmU. Arch Microbiol 193: 751–757. 10.1007/s00203-011-0715-8 21594607

