# Supplementary material for: Depletion of M. tuberculosis GlmU from Infected Murine Lungs Effects the Clearance of the Pathogen
Source: PLoS Pathog. 2015 Oct 21;11(10):e1005235. doi: 10.1371/journal.ppat.1005235 (PMC4619583; doi:10.1371/journal.ppat.1005235)
Supplement: S1 Text — Designing and development of allosteric site inhibitors of GlmUMtb. Screening of inhibitors. Isothermal Titration Calorimetry. Docking and molecular dynamics simulations. H-bond analysis. Survival curve and maximum dose tolerance. Estimation of Oxa33 from treated mice lungs. (DOC) [file ppat.1005235.s001.doc]

**Supmentary Information**

**Supplementary Methods and Results.**

**Designing and development of allosteric site inhibitors of GlmU_Mtb_**

In the present study, we aimed at the design and synthesis of GlmU_Mtb_ allosteric site inhibitors and their efficacy and specificity towards the protein. For this purpose the crystal structure of GlmU_HI_ complexes with a small-molecule inhibitor (PDB code 2VD4) at its allosteric site was considered as the starting point. The design methodology and the workflow are shown in S5A Fig. The GlmU_HI_ inhibitor, 4-chloro-N-(3-methoxypropyl)-N-[(3s)-1-(2-phenylethyl)piperidin-3-yl]benzamide, was found to be interacting with Glu224 and also involved in hydrophobic interactions with Val131, Leu133, Tyr139, Val168, Val220, Val223 and Ala234 (S4A and S4B Fig). Shape based modelling using this inhibitor was carried out employing ROCS methodology. The model generated was able to reproduce the same features which were seen in the crystal structure interactions. This model was used as a query and a ROCS run was performed for screening the Asinex database (ASINEX Platinum and Gold Collection) using ImplicitMillsDean force field to map all the compounds to the shape model. A total of 25,000 hits were resulted from the ROCS screening from which 10,000 hits were carried forward for molecular docking studies which were shortlisted based on the EON scoring. The molecular docking workflow operated, using Glide, included the docking of compounds in both standard precision (SP) and extra precision (XP) modes. The XP docking resulted in 126 compounds, of which 43 hits were selected based on glide score, interaction pattern and tanimoto scores and procured from to perform their inhibition studies against GlmU_Mtb_ (S5A Fig).

**Screening of inhibitors**

Among the 43 compounds screened, one molecule (4*Z*)-4-(2-fluorobenzylidene)-2-(naphthalen-2-yl)-1,3-oxazol-5(4*H*)-one, inhibited ~90% GlmU_Mtb_ uridyltransferase activity at 100 µM (S5A Fig). The compound was further structurally optimized by increasing the hydrophobic component over the phenyl moiety by various scaffolds thereby synthesizing a library of 53 compounds which were in turn subjected to GlmU_Mtb_ enzyme inhibition studies (S5A Fig). Among the 53 compounds, (4*Z*)-4-(4-benzyloxybenzylidene)-2-(naphthalen-2-yl)-1,3-oxazol-5(4*H*)-one (Oxa33), found to inhibit GlmU_Mtb_ efficiently with an IC_50_ of 9.96±1.1 µM (Fig 7A &B).

**Isothermal Titration Calorimetry**

Binding parameters of Oxa33 to GlmU_Mtb_ were carried out by ITC experiments. Binding isotherm (fitted for one site binding) suggests that Oxa33 binds to GlmU_Mtb_ protein with adequately high binding affinity (*K_a_* = 2.35×10^6^ M^-1^) and binding stoichiometry was found n = 0.7. This binding was mainly entropy driven with *TΔS* = 5.8 kcal/mol and exothermic enthalpy change *ΔH* = -2.8 kcal/mol (S6A Fig).

**Docking and Molecular dynamics simulations**

The docking studies of Oxa33 compound were carried out in order to identify its mode of binding at the pocket. Oxa33 was found to be interacting strongly with the active site residues at the GlmU_Mtb_ allosteric site by means of polar and non-polar interactions (S6B and S6C Fig). The oxygen linking the phenyl group with benzyl was found to be in hydrogen bonding with Tyr150 and the carbonyl oxygen over the oxazole ring was involved in hydrogen bonding with Glu250 and Arg253 (S6C Fig). Also, the compound seemed to be well stabilized by strong hydrophobic interactions with Leu144, Pro147, Phe148, Tyr150, Ala233, Ala236 and Leu247.

Molecular dynamics simulations were carried out explicitly for GlmU_Mtb_ complex with Oxa33 at its allosteric site for a period of 20 ns using OPLS_2005 force field of Desmond. The trajectory analysis was carried out so as to study the stability of the complex. The root mean square deviation for all the atoms was plotted against the time scale (S6B Fig). From the plot, it can be inferred that in spite of the fluctuations observed during the initial time period, which can be attributed for the relaxation of the model in the solvent system, the complex was found to be stable during the remaining time scale. This signifies the stabilization effect of the compound over GlmU_Mtb_ and its better binding affinity with the protein.

**H-bond analysis**

Hydrogen bonding, being one of the crucial inter atomic interactions, helps in retaining the bound molecule at the protein active site thereby accounting for its bonding affinity towards the protein. In order to study the hydrogen bond network of GlmU_Mtb_ -Oxa33 complex and its stability during simulation, hydrogen bond analysis for the trajectory was carried out which can in turn be correlated to the GlmU_Mtb_ inhibitory activity. Oxa33 was found to be in hydrogen bonding with Tyr150, Glu250 and Arg253 residues. The inter-atomic distance fluctuations between the compound and these residues were monitored during the simulation and were plotted against time period (S6C Fig). From the plot, it can be inferred that the hydrogen bonding with Glu250 was found to be stable as there were least fluctuations observed when compared to the remaining two. The bonding with Tyr150 and Arg253 continued to be stable throughout the simulation period in spite of some minor fluctuations. These fluctuations of the residues can be well correlated with respect to their crystallographic B factor whose values are ~26, ~21 and ~31 for residues Tyr150, Glu250 and Arg253 respectively. The stability of Glu250 during simulation can be well explained by its lower B factor.

The GlmU_Mtb_ allosteric site residues interacting with oxa33 were mutated and the mutated GlmU_Mtb_ activity was studied in the absence and presence of Oxa33. Residues Tyr150 which was found to be involved in both polar and non-polar interactions with oxa33 was mutated to phenyl alanine (aromatic amino acid) and alanine. Similarly, Gln253 was mutated to glutamine and alanine. Leu144, Gln243 and Leu247 involved in hydrophobic interactions were mutated to alanine. The conventional GlmU_Mtb_ activity, in the absence of Oxa33, was found to be unaffected by these mutations with an exception of L247A which resulted in increase in activity by 50%, shown in (Fig 7D). Also, Y150A resulted in quiet lower decrease in activity, while Y150F being unchanged, signifying the importance of an aromatic amino acid at 150^th^ position for activity. The mutated GlmU_Mtb_ activity studies in the presence of Oxa33 resulted in quite interesting yet significant results (Fig 7E). Mutations Y150F, Y150A and L247A resulted in complete loss of Oxa33 activity, which IC_50_ was found to be greater than 500 µM when compared to the GlmU_Mtb_ -Oxa33 activity (IC_50_ ~10 µM). This loss in Oxa33 activity can be accounted for its inability to interact with the mutated residues at the allosteric site, which in turn signifies the binding ability of the compound to the GlmU_Mtb_. Mutation of Arg253 to glutamine and alanine resulted in nearly 8 to 9 fold decrease in Oxa33 activity. Other mutations, Q253A and L144A, did not affect the activity specifying their lesser significance in the Oxa33 binding.

**Survival curve and maximum dose tolerance**

Different groups of BALB/c mice (n=4 mice per group) were treated with various concentrations of Oxa33 inhibitor (25 mg/kg, 50 mg/kg, 100 mg/kg and 200 mg/kg) resuspended in 2.5% Tween-80) through intra-peritoneal route for 30 days on alternate day. Observations were taken as relative change in body weight and survival of mice and plotted as function of time in GraphPad Prism 5.0 (S8A and S8B Fig).

**Estimation of Oxa33 from treated mice lungs**

Tetrahydrofuran (THF) was found to be best solvent after checking the solubility of Oxa33 in various organic solvents. UV spectrophotometric based wave scan analysis confirmed the λ_max_ of Oxa33 at 401 nm in THF. A standard curve (ranging from 2.5 µg/ ml to 40 µg/ ml of Oxa33) was prepared by measuring absorbance at 401 nm (S10A and S10B Fig). BALB/c mice were treated with Oxa33 (50 mg/kg in 2.5% Tween-80) for 3 weeks and 8 weeks and the lungs were isolated. Oxa33 was extracted from the lungs macerated lungs (using Dounce homogenizer) and the absorbance of 10 time diluted sample was measured at 401 nm (S10C Fig). The amount of Oxa33 in the lungs was calcuated with the help of slope obtained from the standard curve.
