# Supplementary figures and images for: Depletion of M. tuberculosis GlmU from Infected Murine Lungs Effects the Clearance of the Pathogen
Source: PLoS Pathog. 2015 Oct 21;11(10):e1005235. doi: 10.1371/journal.ppat.1005235 (PMC4619583; doi:10.1371/journal.ppat.1005235)

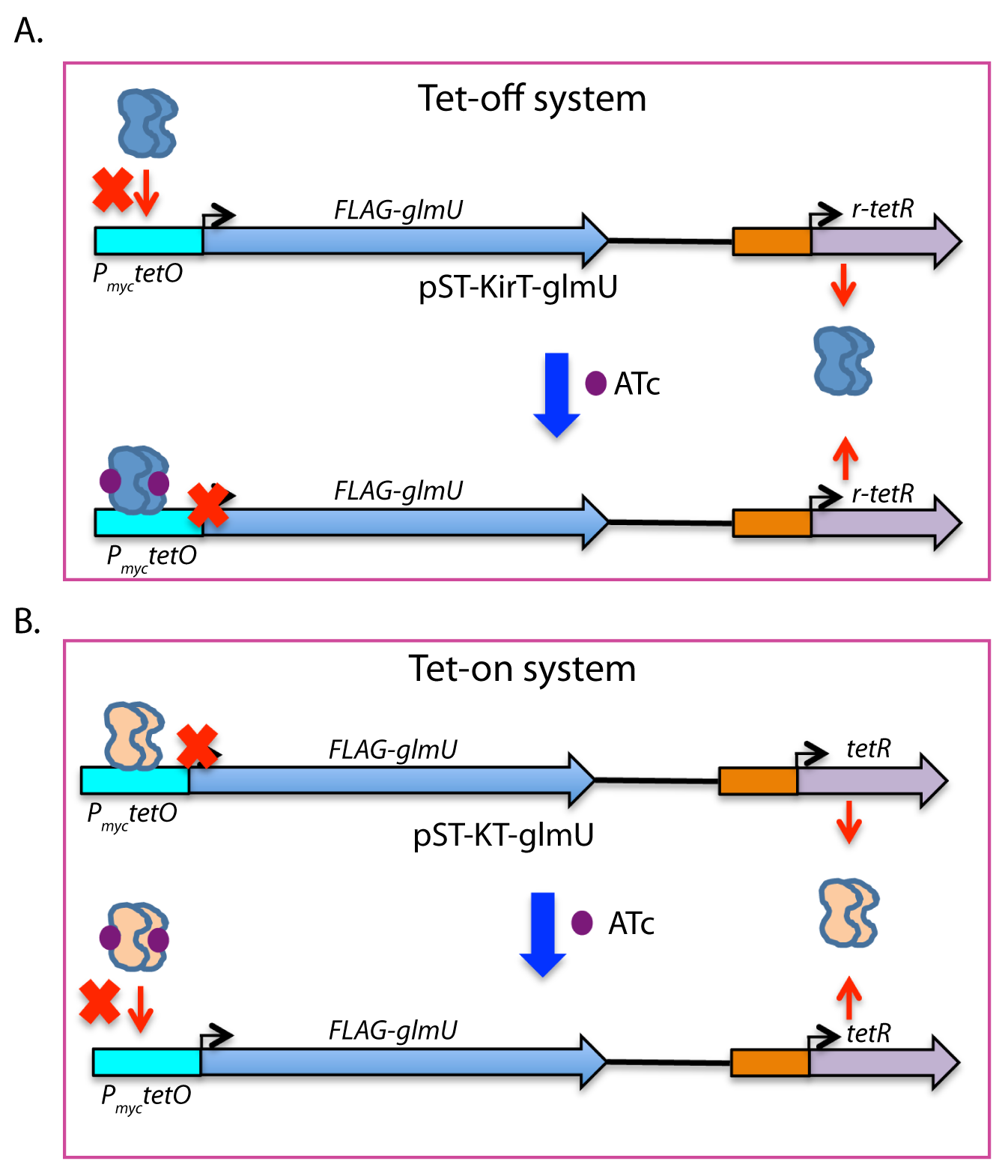

Supplement: S1 Fig — (TIF) [file ppat.1005235.s002.tif]

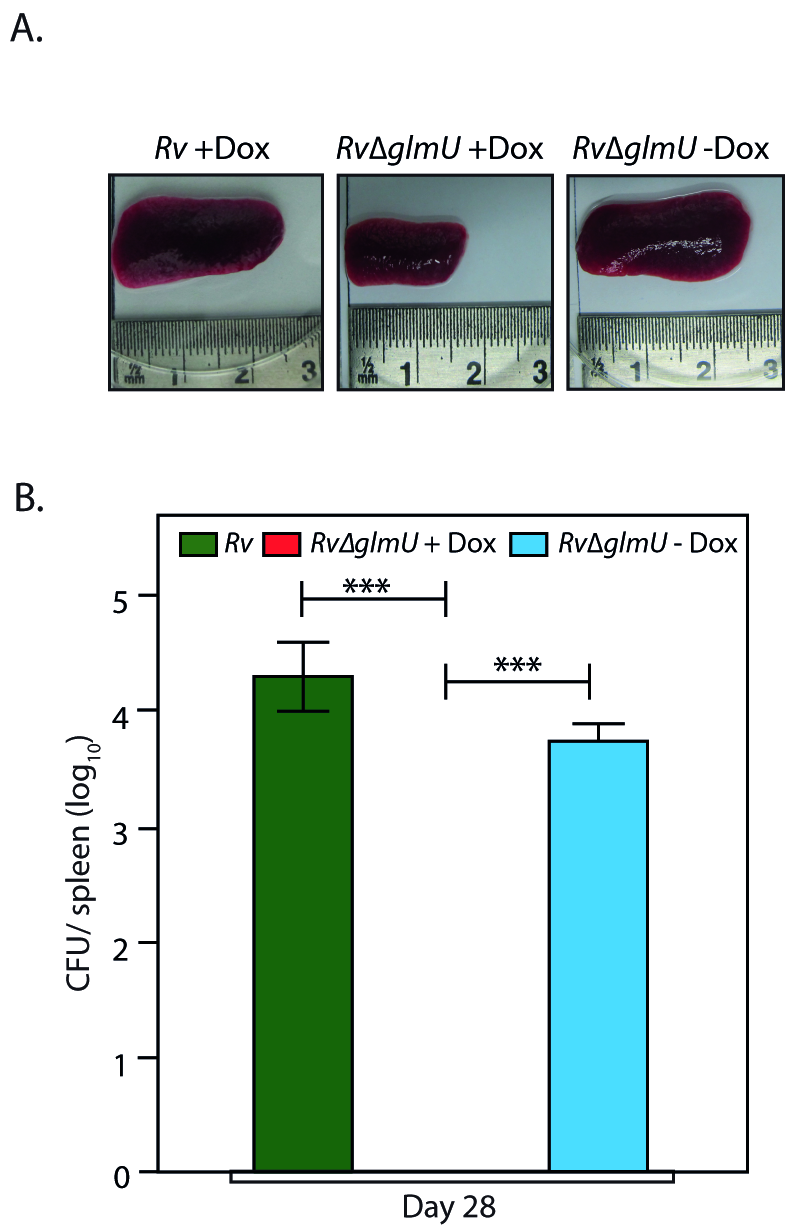

Supplement: S2 Fig — Guinea pigs (six per group) were infected with Rv or Rv∆glmU and Dox was provided in the water as indicated in Fig 5. (A) Overall pathology of the infected spleens from the guinea pigs 4 weeks post infection. (B) CFU data from guinea pig spleens. ***p<0.0001 or, two tailed non parametric t-test, mean, error bars indicate s.e.m. (TIF) [file ppat.1005235.s003.tif]

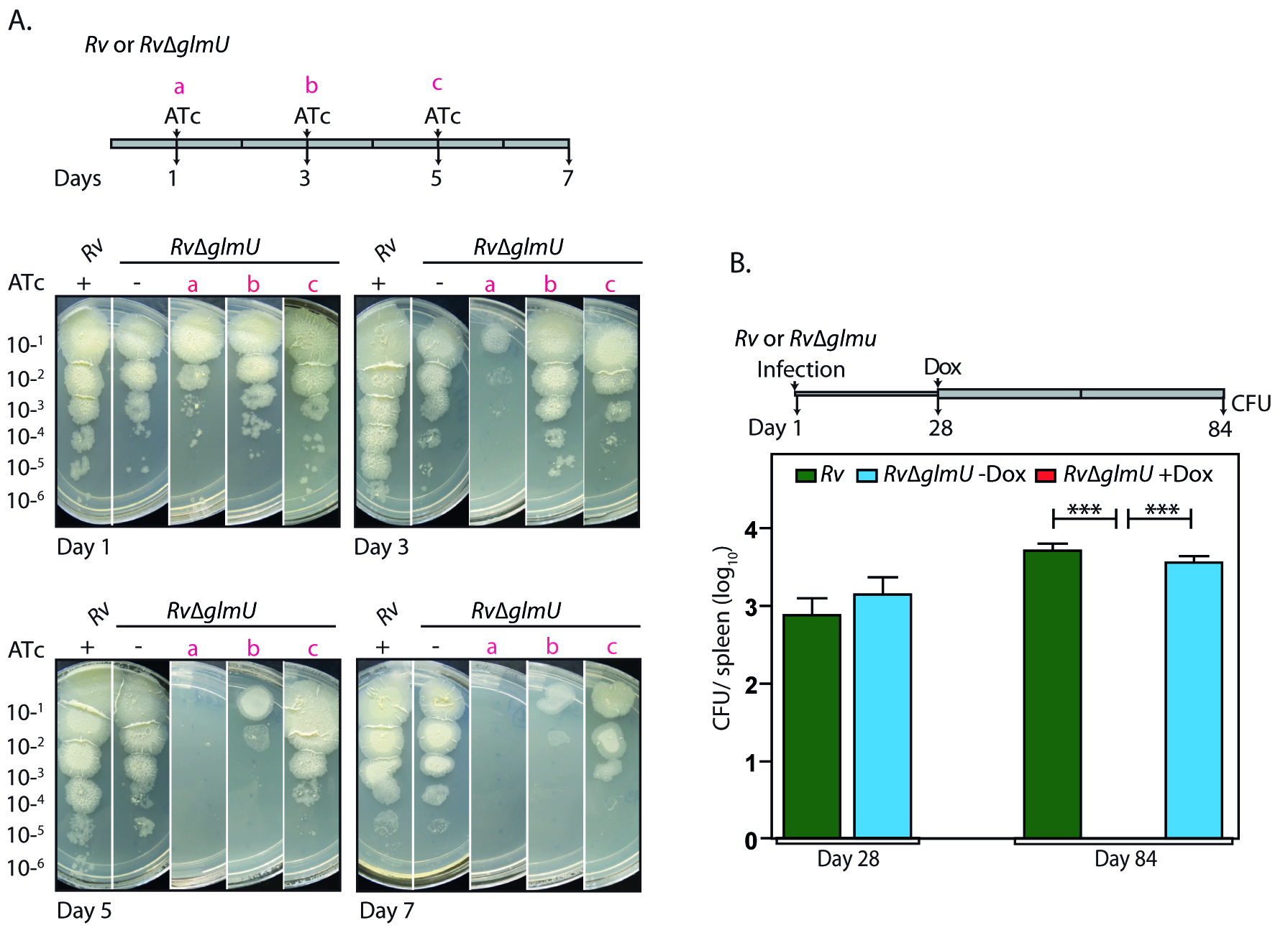

Supplement: S3 Fig — (A) Rv and Rv∆glmU cultures were inoculated at an initial A 600 of 0.1. ATc was added to the Rv culture on day 1 (a) and Rv∆glmU cultures were either grown in the absence of ATc or was supplemented with ATc in the growth media on 1st (a), 3rd (b) or 5th (c) day. Serially diluted cultures were spotted on 7H11 agar plates after day 1, 3, 5 and 7. The experiment was performed in triplicates. (B) BALB/c mice (6 to 9 / group) were infected with Rv and Rv∆glmU strains. 28 days post infection Dox was provided for Rv and Rv∆glmU infected mice and one group of Rv∆glmU infected mice were administered with the vehicle control for the next 56 days. CFUs were enmurated from the spleens of infected mice on day 28 and day 84 post infection. At 28 days post infection mean CFUs for the spleens of Rv and Rv∆glmU infected mice were 2.9 and 3.15 on log10 scale and 84 days post infection mean CFUs for Rv +Dox or Rv∆glmU +Dox and Rv∆glmU–Dox infected spleens were 3.67, 0 and 3.5 on log10 scale. ***p<0.0005, two tailed non parametric t-test, error bars indicate s.e.m. (TIF) [file ppat.1005235.s004.tif]

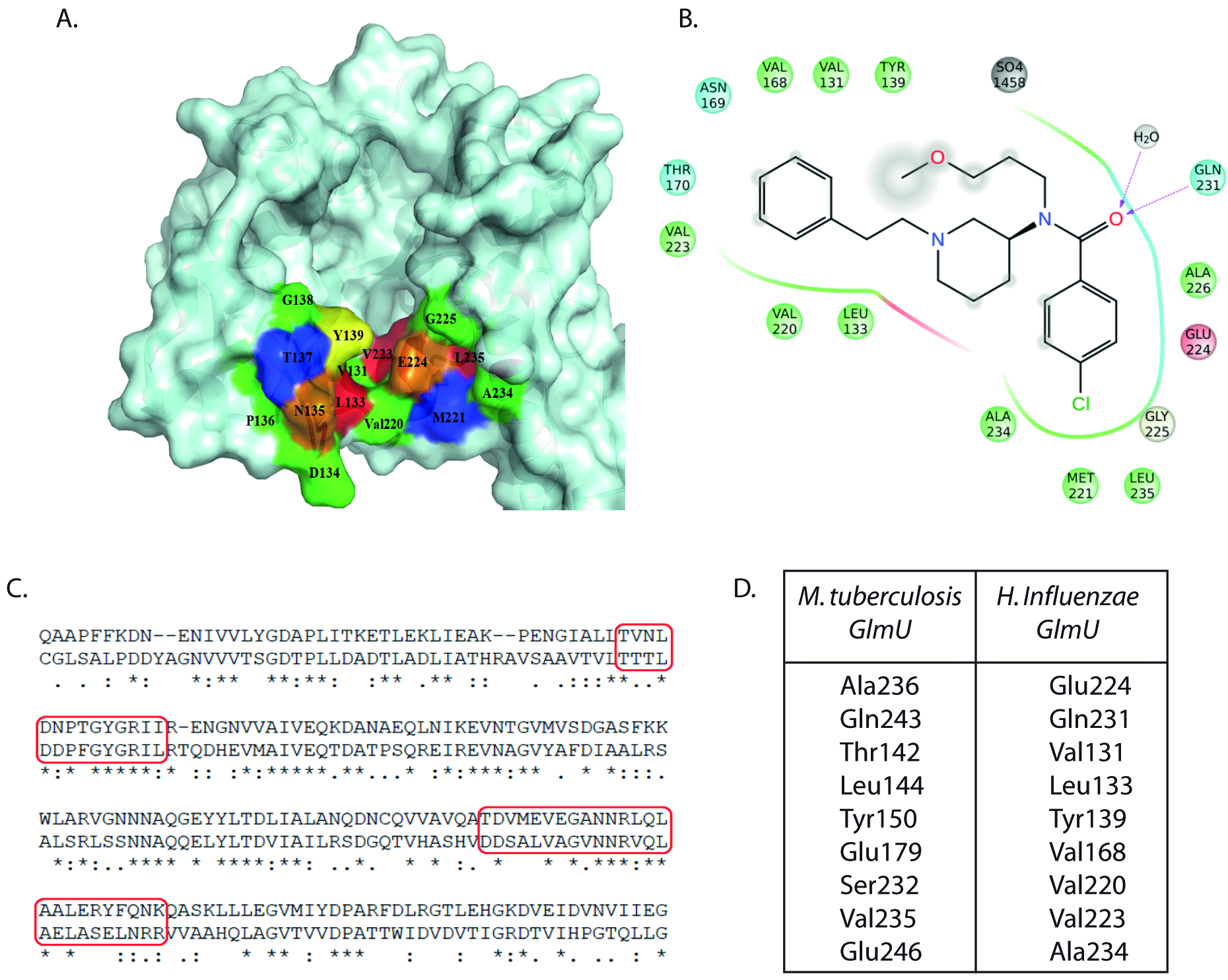

Supplement: S4 Fig — (A) Surface representation of GlmUHI wherein its allosteric residues are highlighted. The allosteric site of GlmUHI, comprises of highly lipophilic surface and a distal depression surface. (B) Interction of GlmUHI allosteric inhibitor at the allosteric site. Hydrophobic residues are coloured in green, polar residues in blue, negatively charged residues in red. Hydrogen bond interaction of the ligand with Gln231 is shown in pink color; Glu224 is also involved in the hydrogen bonding which is not represented here. Leu133, Tyr139, Met221, Leu235 were found to be involved in strong hydrophobic interactions with the inhibitor. Interactions were plotted with LIGPLOT. (C) Sequence alignment of GlmUHI and GlmUMtb allosteric site residues. Conserved residues are highlighted in red boxes. (D) Table displaying allosteric residues of GlmUHI and GlmUMtb. Some of the critical residues such as Tyr150, Gln243 and Leu247 of GlmUMtb are seen to be conserved. (TIF) [file ppat.1005235.s005.tif]

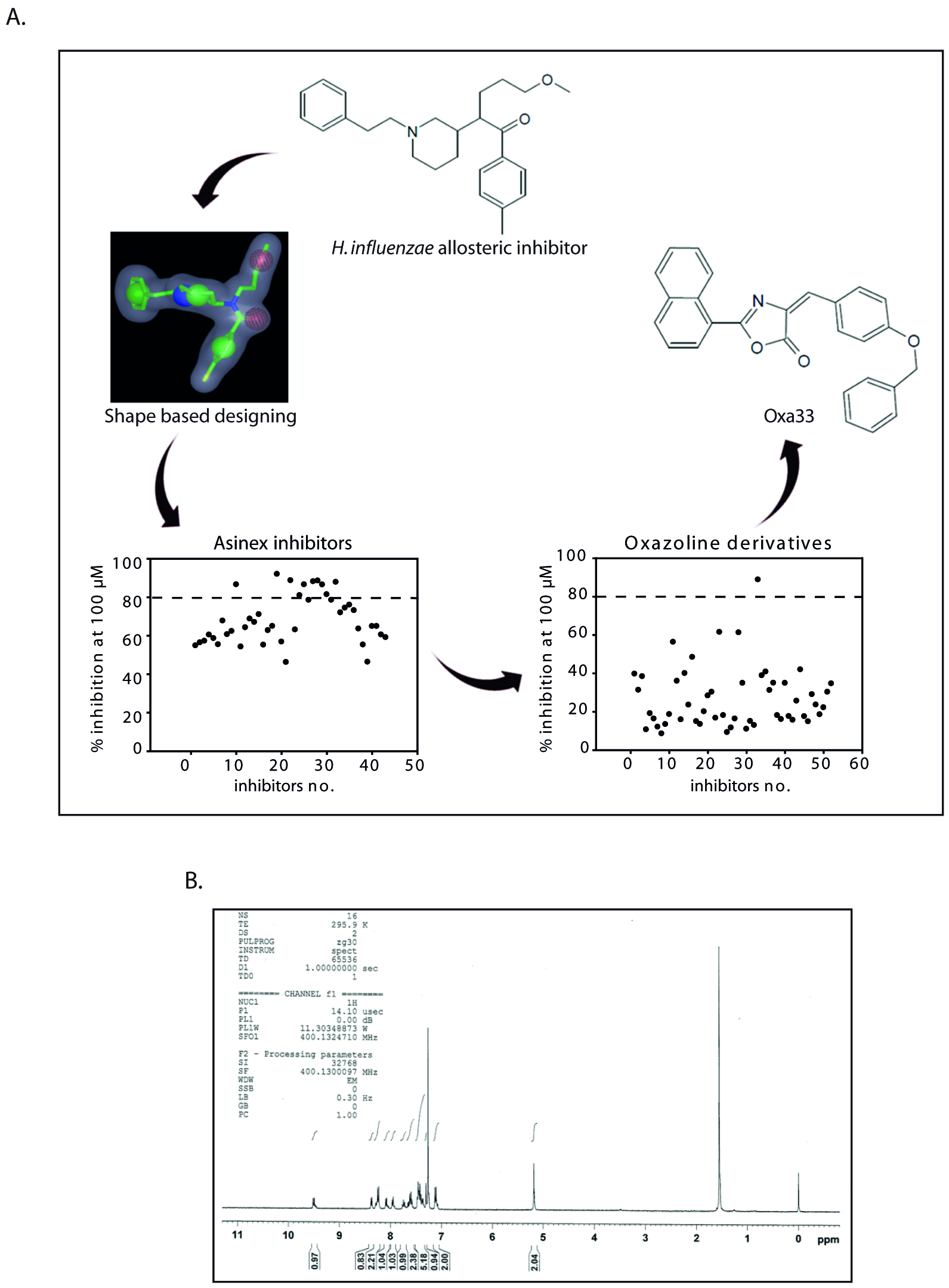

Supplement: S5 Fig — (A) Virtual screening workflow used towards the identification of allosteric inhibitors for GlmUMtb. Shape model of allosteric inhibitor of GlmUHI was generated using ROCS software. In the initial set of screening, a total of 43 compounds were identified and tested for their ability to inhibit uridyltransferase reaction. 10 among the 43 compounds showed ~90% inhibition at 100 μM. Of these, an oxozolone derivative was identified and further chemically derivatized to a library of 52 compounds. One compound from these, Oxa33, which showed ~90% inhibition was considered for further studies. (B) Nuclear magnetic resonance spectra of purified Oxa33 (Yellow solid). 1H NMR (400 MHz, CDCl3): δ 9.51 (d, J = 8.8 Hz, 1H), 8.38 (d, J = 8.8 Hz, 1H), 8.25 (d, J = 8.4 Hz, 2H), 8.10 (d, J = 8.4 Hz, 1H), 7.93 (d, J = 8.0 Hz, 1H), 7.74 (t, J = 8.0 Hz, 1H), 7.68–7.56 (m, 2H), 7.49–7.33 (m, 5H), 7.29 (s, 1H), 7.12 (d, J = 8.4 Hz, 2H), 5.15 (s, 2H). Anal calcd for: C27H19NO3: C, 79.98; H, 4.72; N, 3.45% Found C, 79.92; H, 4.83; N, 3.54%. (TIF) [file ppat.1005235.s006.tif]

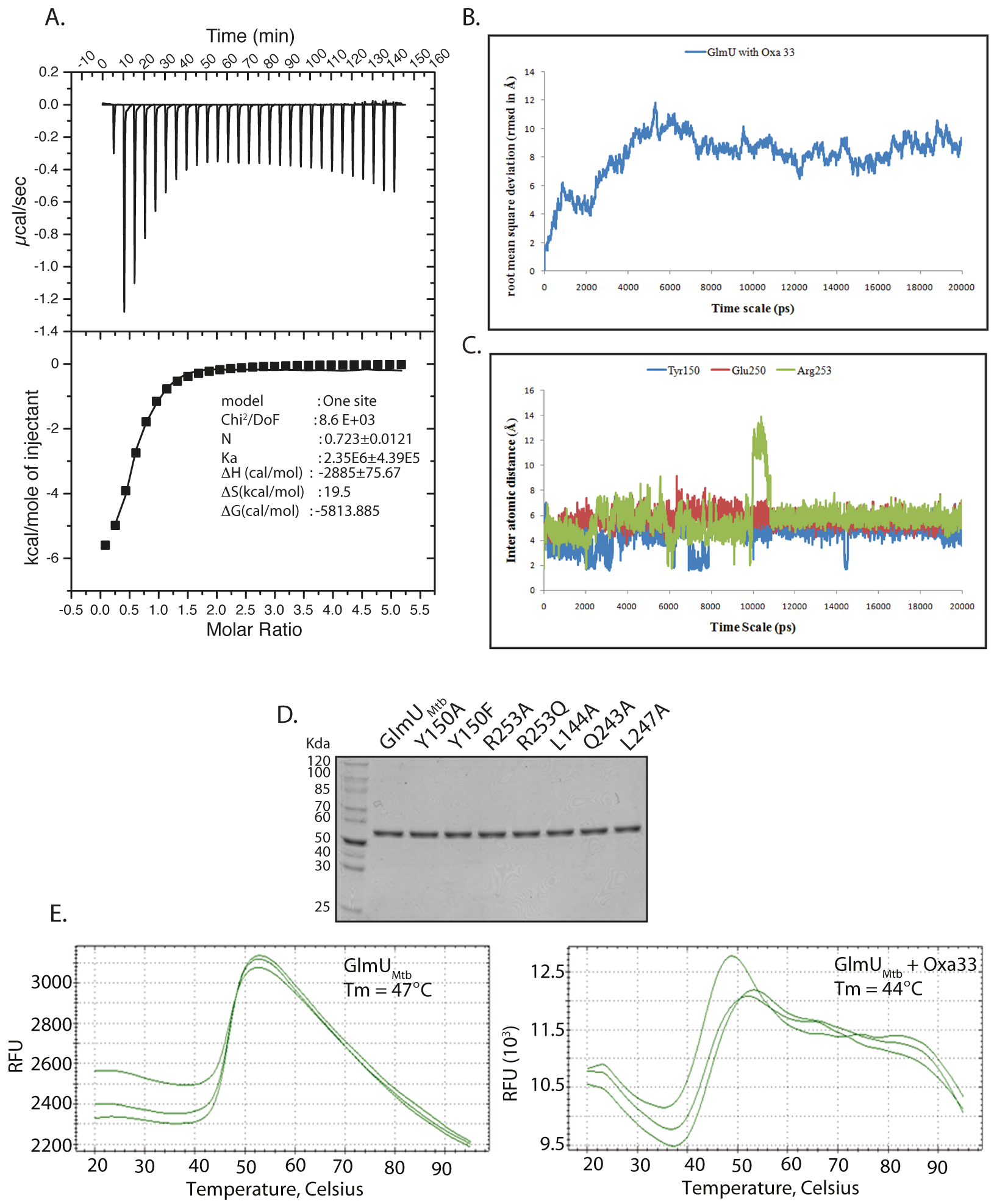

Supplement: S6 Fig — (A) Isothermal titration calorimetry results of Oxa33 with GlmUMtb. Released heat increased over the period of time (μcal/sec) is presented in upper panel while corresponding binding isotherm (fitted for one site) presented in lower panel. (B) rmsd plot for GlmUMtb-oxa33 complex simulated for a period of 20ns. The complex was found to be unstable during the initial time period which can be owed for its relaxation. After 8 ns, the complex was observed to be stable indicating the strong binding affinity of Oxa33 towards GlmUMtb (C) Hydrogen bond analysis of Oxa33 analyzed during the simulation time period. (D) Purified GlmUMtb mutants (single band) specified by the docking studies of Oxa33. (E) Differential Scanning Fluorimetry (DSF; performed in triplicates) results of Oxa33 with GlmUMtb. Left panel representing melting curve of GlmUMtb in presence of 5% DMSO. While right graph is showing -3°C Tm shift upon Oxa33 incubation with GlmUMtb protein. (TIF) [file ppat.1005235.s007.tif]

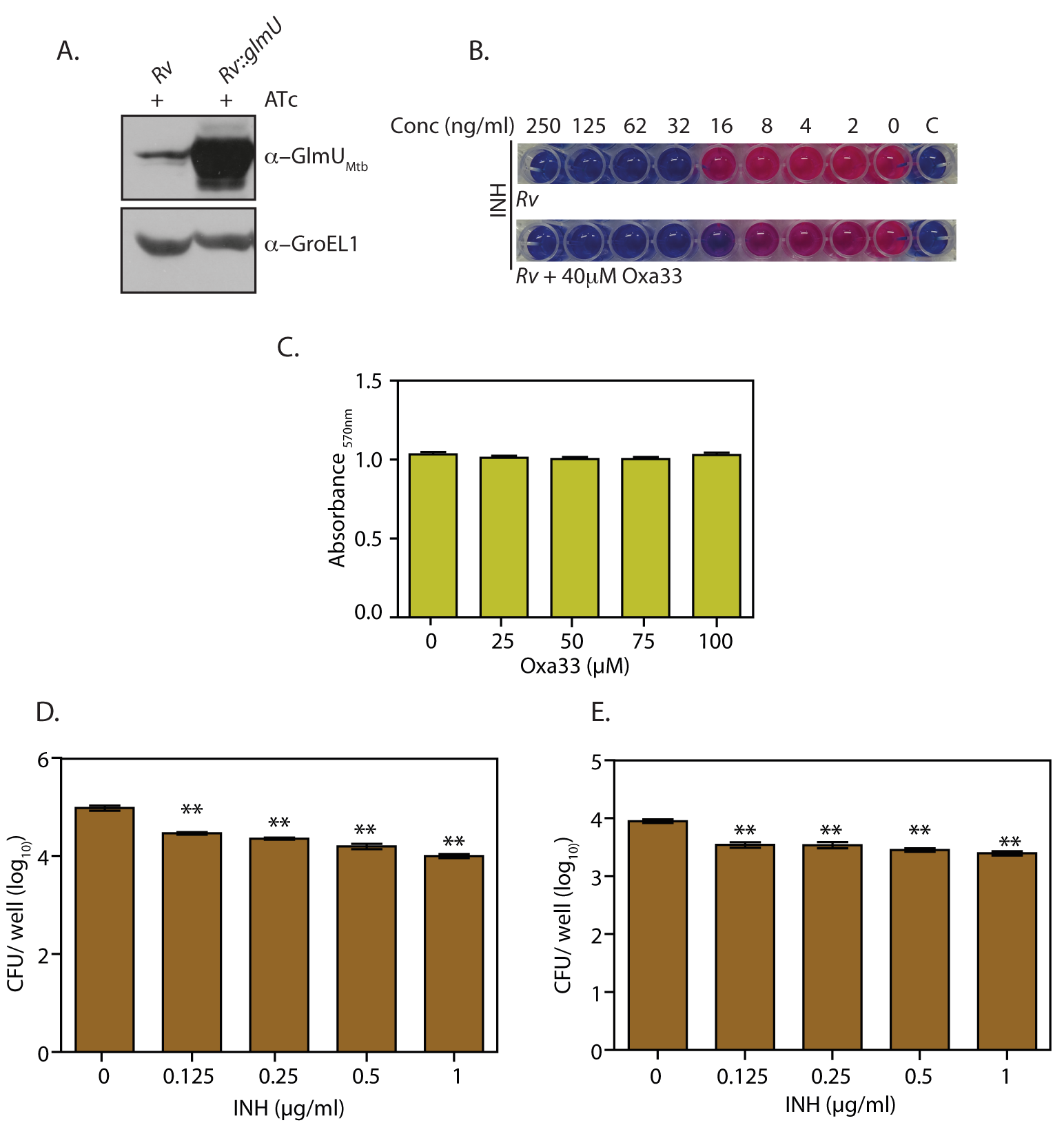

Supplement: S7 Fig — (A) Rv and Rv::glmU tet-on strains were inoculated at an initial A 600 of 0.1 in the presence of ATc (2 μg/ml) and grown up to 5 days. WCLs were resolved and probed with anti-GlmU and anti-GroEL1 antibodies. (B) Rv culture was treated with sub lethal dose of Oxa33 (40 μM) and variable concentrations of INH for 6 days followed by resazurine addition. Results show decrease in MICs of INH from 32 ng/ml to 16 ng/ml. The experiment was pefromed in triplicates. (C) Bar graph presents the cytoxicity of the Oxa33 on THP1 cells during the 3 days of treatment. Cell viability was checked with alamar blue based assay and absorbance at 570 nm was plotted with increasing concentrations of Oxa33 inhibitor. The experiment was performed in triplicates and the error bars represent s.e.m. (D) CFUs count results of THP1 cells infected with Rv (for 24 h post infection) followed by treatment with various concentrations of INH (in water) for 3 days. CFU (log10) per well shows gradual increase in bacterial death with increasing INH doses. **p<0.005, two tailed non parametric t-test, mean, s.e.m., n = 3). (E) Graph illustrate CFU numbers from fully infected THP1 cells (24 h) with Rv:: glmU tet-on. Abundant GlmUMtb expression in Rv::glmU tet-on infected THP1 cells (with ATc) does not affect INH efficacy, which gives similar results as Rv. **p<0.005, two tailed non parametric t-test,. error bars represent s.e.m. (TIF) [file ppat.1005235.s008.tif]

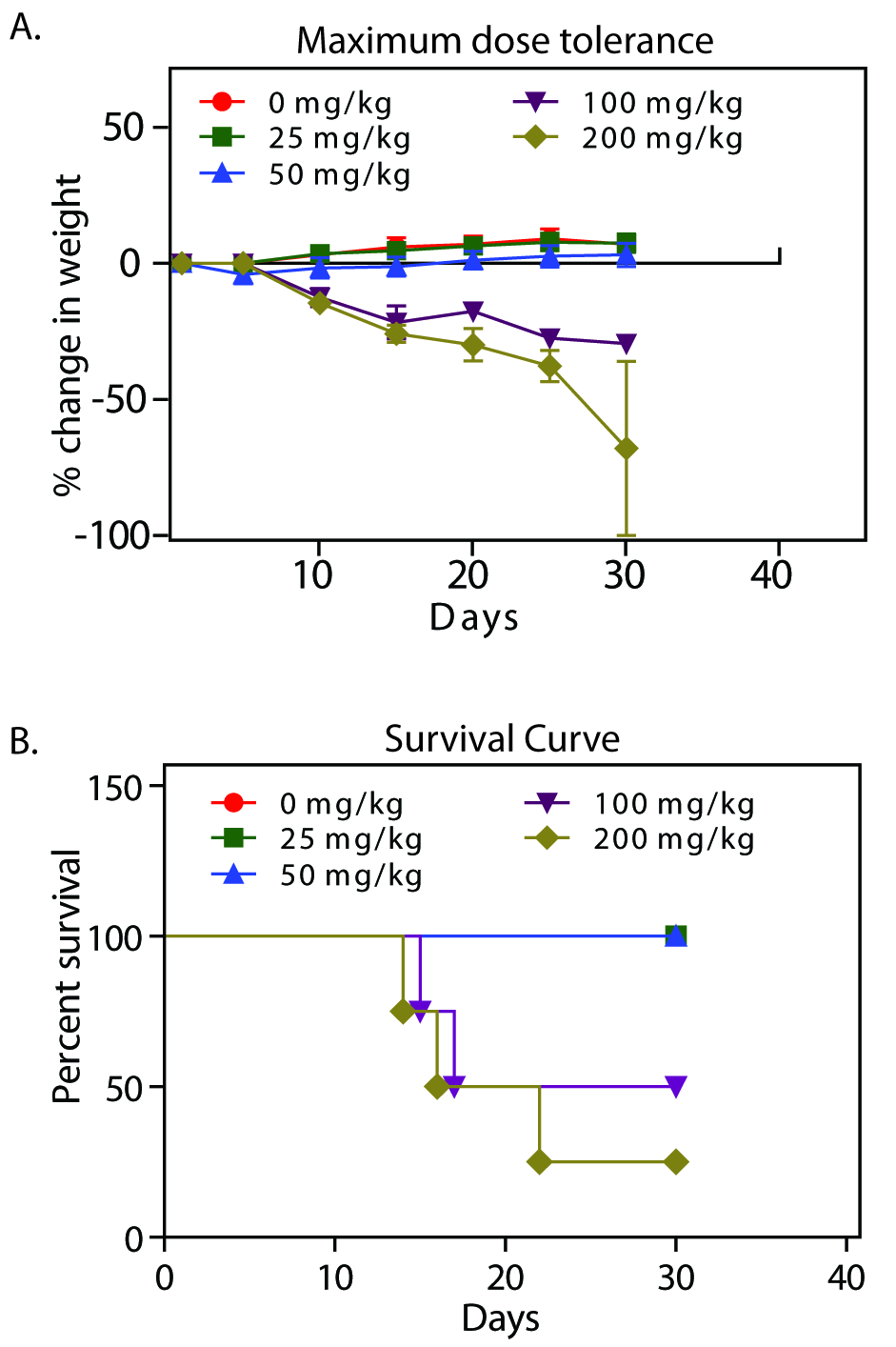

Supplement: S8 Fig — (A) Maximum dose tolerance graph showing relative change in body weight of mice (4 mice / group) during the course of Oxa33 administration for 30 days. (B) Survival curve of mice (4 mice / group) treated with different concentrations of Oxa33 for 30 days. (TIF) [file ppat.1005235.s009.tif]

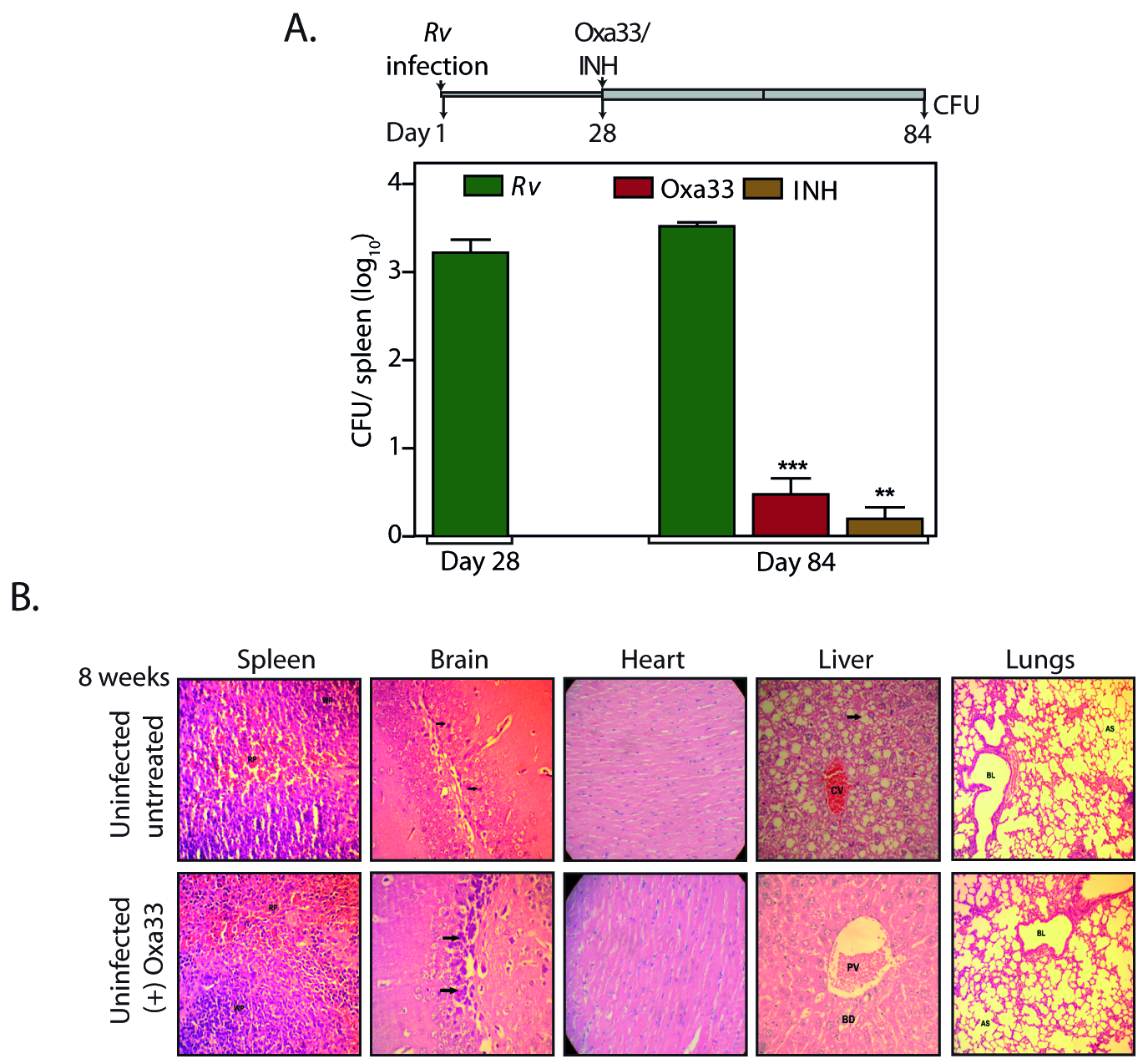

Supplement: S9 Fig — (A) Mtb bacterial load in the spleen of totally infected mice (7 to 11 mice /group) (for 28 days) and treated with Oxa33 or INH for subsequently 56 days. ***p<0.0007 or **p<0.002, two tailed non parametric t-test, the error bars represent s.e.m. (B) High power (400x) photomicrograph of hematoxylin and eosin stained spleen, brain, heart, liver and lungs of untreated mice or mice treated with Oxa33 for 8 weeks. Spleen is showing usual parenchyma. WP = White Pulp, RP = Red Pulp. While, section from hippocampal area of brain showing several degenerated neuron in the neuronal layer. Section of heart is showing normal cardiac muscle fibres. Photomicrograph of liver depicts typical hepatic parenchyma. PV = Portal Vein, BD = Bile Duct. Lung section also represents usual lung parenchyma. BL = Bronchial Lumen, AS = Alveolar space. (TIF) [file ppat.1005235.s010.tif]

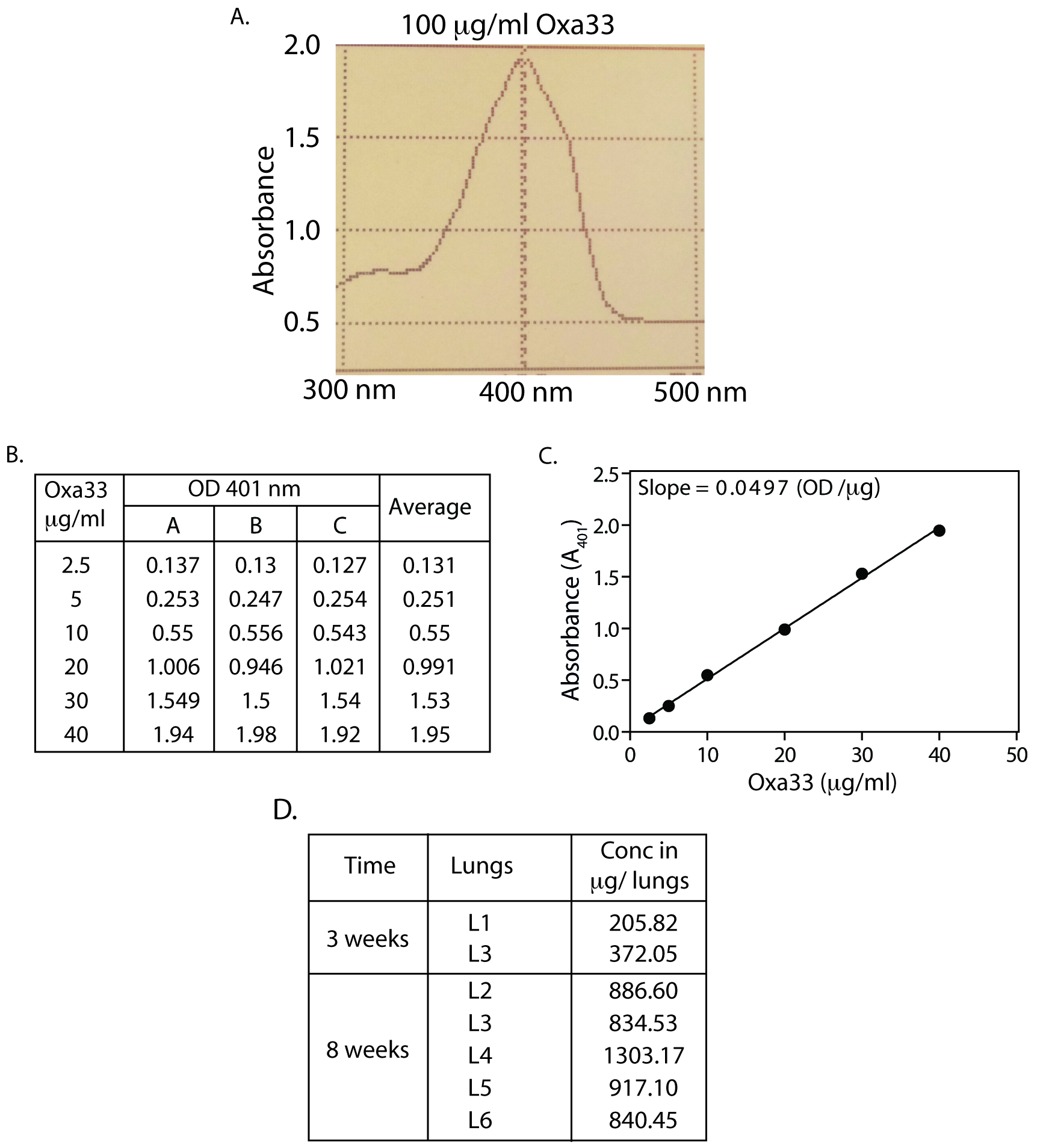

Supplement: S10 Fig — (A) Absorbance spectra of Oxa33 resuspended in tetrahydrofuran (THF). It showed a peak at 401 nm. (B) Absorbance readings (401 nm; performed in triplicates; A, B and C) of Oxa33 at different concentrations (C) Standard curve of Oxa33 showing linear regression with a slope of 0.0497 OD / μg. (D) Table showing Oxa33 concentrations (μg/lung) in the lungs of mice treated with 50 mg /kg (body weight) for 3 or 8 weeks. (TIF) [file ppat.1005235.s011.tif]

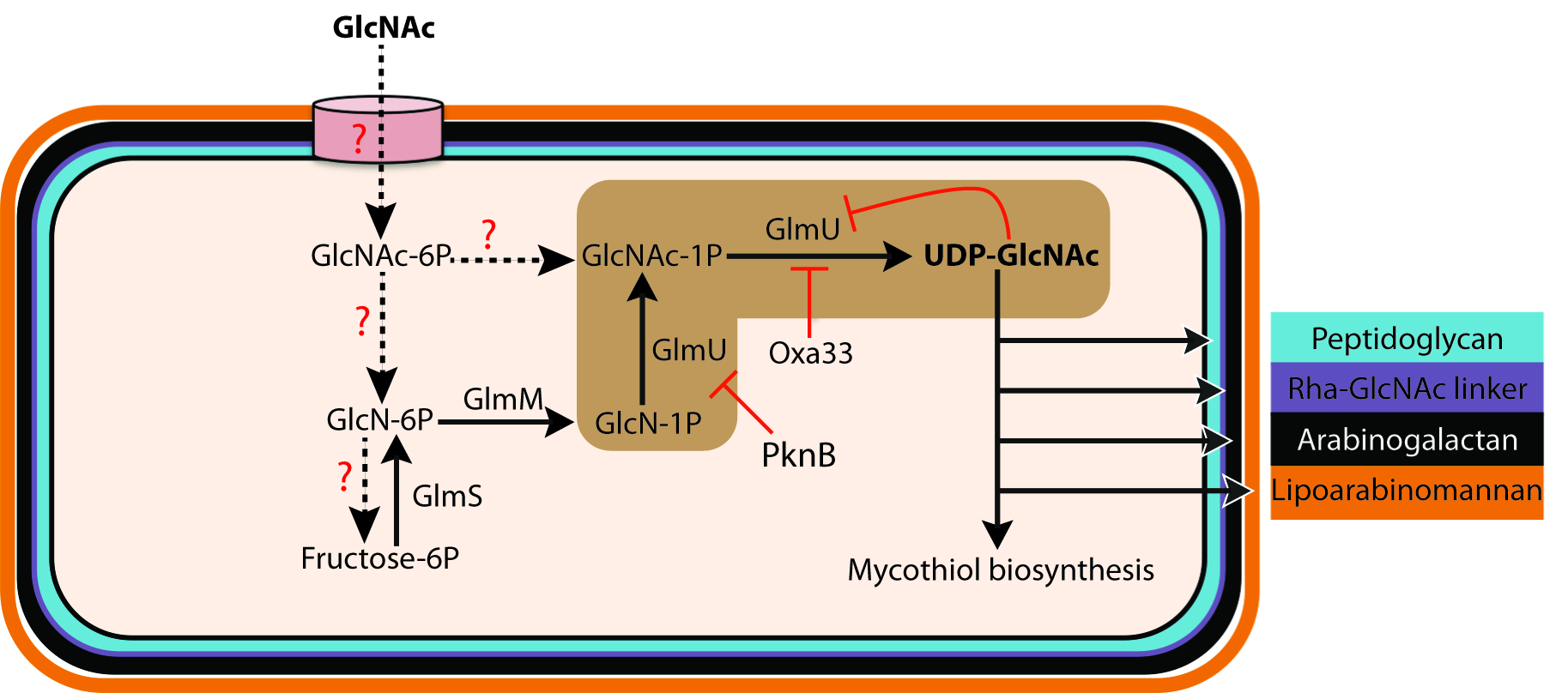

Supplement: S11 Fig — Model shows De novo pathway for UDP-GlcNAc synthesis is mediate by GlmS, GlmM and GlmU enzymes. Shaded pathway is conserved in Mtb. UPD-GlcNAc can inhibit uridyltransferase activity by feedback inhibition mechanism. Also GlcNAc from host resources or from cell wall recycling can be transported inside the bacteria and further metabolized and feeded into the de novo pathways through GlmS/ GlmU mediated reactions. Question marks show that these pathways are still not characterized in Mtb. Dashed lines shows possible input of substrates or unknown pathway while complete lines shows established and known pathways for UDP-GlcNAc synthesis. (TIF) [file ppat.1005235.s012.tif]
