# Supplementary material for: Depletion of M. tuberculosis GlmU from Infected Murine Lungs Effects the Clearance of the Pathogen
Source: PLoS Pathog. 2015 Oct 21;11(10):e1005235. doi: 10.1371/journal.ppat.1005235 (PMC4619583; doi:10.1371/journal.ppat.1005235)
Supplement: S1 Table — (DOC) [file ppat.1005235.s013.doc]

**S1 Table. List of bacterial strains, plasmids and phages used in the study**

| **Plasmid constructs** | **Description** | **Source** |
| --- | --- | --- |
| pQE2 | *E. coli* expression vector, T5 promoter, Amp^R^ | Qiagen |
| pQE2-His_6_-*glmU_Mtb_* | His_6_ tag *glmU_Mtb_* cloned into *NdeI*/*HindIII* sites of MCS | This study |
| pQE2-His_6_-*glmU*_1-352_ | His_6_ tag *glmU*_1-352_ cloned into *NdeI*/*HindIII* sites of MCS | This study |
| pQE2-His_6_-*glmU*_150-495_ | His_6_ tag *glmU*_150-495_ cloned into *NdeI*/*HindIII* sites of MCS | This study |
| pQE2-His_6_-*glmU*_K26A_ | His_6_ tag *glmU*_K26A_ cloned into *NdeI*/*HindIII* sites of MCS | This study |
| pQE2-His_6_-*glmU*_H374A_ | His_6_ tag *glmU*_H374A_ cloned into *NdeI*/*HindIII* sites of MCS | This study |
| pQE2-His_6_-*glmU*_DM_ | His_6_ tag *glmU*_DM_ cloned into *NdeI*/*HindIII* sites of MCS | This study |
| pQE2-His_6_-*glmU*_Y150A_ | His_6_ tag *glmU*_Y150A_ cloned into *NdeI*/*HindIII* sites of MCS | This study |
| pQE2-His_6_-*glmU*_Y150F_ | His_6_ tag *glmU*_Y150F_ cloned into *NdeI*/*HindIII* sites of MCS | This study |
| pQE2-His_6_-*glmU*_L247A_ | His_6_ tag *glmU*_L247A_ cloned into *NdeI*/*HindIII* sites of MCS | This study |
| pQE2-His_6_-*glmU*_R253A_ | His_6_ tag *glmU*_R253A_ cloned into *NdeI*/*HindIII* sites of MCS | This study |
| pQE2-His_6_- *glmU*_R253Q_ | His_6_ tag *glmU*_R253Q_ cloned into *NdeI*/*HindIII* sites of MCS | This study |
| pQE2-His_6_-*glmU*_Q242A_ | His_6_ tag *glmU*_Q242A_ cloned into *NdeI*/*HindIII* sites of MCS | This study |
| pQE2-His_6_-*glmU*_L144A_ | His_6_ tag *glmU*_L144A_ cloned into *NdeI*/*HindIII* sites of MCS | This study |
| pTC28S15-OX | Contains reverse tetracycline repressor. | Addgene |
| pST-KirT | Integrative (for *attB* site) *Mtb* expression vector with N-terminal FLAG tag and reverse tetracycline repressor gene (r*tetR)*. | This study |
| pST-KirT-*glmU_Mtb_* | *GlmU_Mtb_* cloned into *NdeI*/*HindIII* sites of MCS of pST-KirT under P_myc1_*tetO* promotor. | This study |
| pSTKT-*glmU_tet-on_* | FLAG tag *glmU* cloned into *NdeI*/*HindIII* sites of MCS under P_myc1_*tetO* promotor. | This study |
| pYUB1474 | Containing the *hyg^R^* antibiotic marker cassette and *oriE + cosλ* | [65] |
| pNIT | *Mtb* expression vector, *nitA* promoter, Chl^R^, IVN inducible | [69] |
| pNIT-*glmU_Mtb_* | *glmU_Mtb_* cloned into *NdeI*/*HindIII* sites of pNIT vector. | This study |
| pNIT-glmU_1-352_ | *glmU*_1-352_ cloned into *NdeI*/*HindIII* sites of pNIT vector. | This study |
| pNIT-*glmU*_150-495_ | *glmU*_150-495_ cloned into *NdeI*/*HindIII* sites of pNIT vector. | This study |
| pNIT-*glmU*_K26A_ | *glmU*_K26A_ cloned into *NdeI*/*HindIII* sites of pNIT vector. | This study |
| pNIT-*glmU*_H374A_ | *glmU*_H374A_ cloned into *NdeI*/*HindIII* sites of pNIT vector. | This study |
| pNIT-*glmU*_DM_ | *glmU*_DM_ cloned into *NdeI*/*HindIII* sites of pNIT vector. | This study |
| **Bacterial strains** | | |
| DH5α | *E. coli* strain, for cloning experiments | Invitrogen |
| BL21 (DE3) codon plus | *E. coli* strain, for protein expression | Stratagene |
| mc^2^155 | Wild type *M, smegmatis* strain | ATCC, 700084 |
| *H37Rv* or *Rv* | Wild type *M. tuberculosis* strain | ATCC |
| *Rv*::*glmU*  (merodiploid strain) | *H37Rv* electroporated with integrative construct pST-KirT-glmU. | This study |
| *Rv*::*glmU_tet-on_* | H37Rv electroporated with episomal pSTKT-*glmU_tet-on_*, containing ATc inducible GlmU_Mtb_, Kan^R^. | This study |
| *Rv*∆*glmU* | H37Rv *glmU* conditional mutant. *glmU_Mtb_* gene expression is under the regulation of ATc inducible Tet promoter, Kan^R^. | This study |
| *Rv*∆*glmU*::pNIT | H37Rv *glmU* conditional mutant strain complimented with pNIT vector only, Chl^R^. | This study |
| *Rv*∆*glmU*::*glmU_WT_* | H37Rv *glmU* conditional mutant strain complimented with pNIT-*glmU_Mtb_*, Chl^R^. | This study |
| *Rv*∆*glmU*::*glmU*_1-352_ | H37Rv *glmU* conditional mutant strain complimented with pNIT-*glmU*_1-352_, Chl^R^. | This study |
| *Rv* ∆*glmU*:: *glmU*_150-495_ | H37Rv *glmU* conditional mutant strain complimented with pNIT-*glmU*_150-495_, Chl^R^. | This study |
| *Rv* ∆*glmU*:: *glmU*_K26A_ | H37Rv *glmU* conditional mutant strain complimented with pNIT-*glmU* _K26A_, Chl^R^. | This study |
| *Rv* ∆*glmU*:: *glmU*_H374A_ | H37Rv *glmU* conditional mutant strain complimented with pNIT-*glmU* _H374A_, Chl^R^. | This study |
| *Rv* ∆*glmU*::*glmU*_DM_ | H37Rv *glmU* conditional mutant strain complimented with pNIT-*glmU* _DM_, Chl^R^. | This study |
| **Phages** | | |
| pHAE159 | Temperature sensitive shuttle phasmid | [66] |
| pHAE159:: *glmU_Mtb_* AES | GlmU_Mtb_ AES constructs were cloned into *PacI* site and used for specialized transduction to replace *glmU_Mtb_* with hygromycin resistance gene in Mtb | This study |
| **His_6_:: 6 Histidine tag; MCS: Multiple cloning site; AES allelic exchange substrate; DM: Double mutant (K26A + H374A); IVN: Iso valeronitrile; ATc: anhydrotratracycline.** | | |
